# Theoretical Investigations on the Reactivity of Methylidyne Radical toward 2,3,7,8-Tetrachlorodibenzo-*p*-Dioxin: A DFT and Molecular Dynamics Study

**DOI:** 10.3390/molecules23102685

**Published:** 2018-10-18

**Authors:** Weihua Wang, Wenling Feng, Wenliang Wang, Ping Li

**Affiliations:** Key Laboratory of Life-Organic Analysis, School of Chemistry and Chemical Engineering, Qufu Normal University, Qufu 273165, China; wlfengqf@163.com (W.F.); wlwangqf@126.com (W.W.)

**Keywords:** methylidyne radical, 2,3,7,8-tetrachlorodibenzo-*p*-dioxin, reaction mechanisms, theoretical calculations

## Abstract

To explore the potential reactivity of the methylidyne radical (CH) toward 2,3,7,8-tetrachlorodibenzo-*p*-dioxin (TCDD), the reaction mechanism between them has been systematically investigated employing the density functional theory (DFT) and *ab initio* molecular dynamics simulations. The relevant thermodynamic and kinetic parameters in the possible reaction pathways have been discussed as well as the IR spectra and hyperfine coupling constants (hfcc’s) of the major products. Different from the reaction of the CH radical with 2,3,7,8-tetrachlorodibenzofuran, CH radical can attack all the C-C bonds of TCDD to form an initial intermediate barrierlessly via the cycloaddition mechanism. After then, the introduced C-H bond can be further inserted into the C-C bond of TCDD, resulting in the formation of a seven-membered ring structure. The whole reactions are favorable thermodynamically and kinetically. Moreover, the major products have been verified by *ab initio* molecular dynamics simulations. The distinct IR spectra and hyperfine coupling constants of the major products can provide some help for their experimental detection and identification. In addition, the reactivity of the CH radical toward the F- and Br-substituted TCDDs has also been investigated. Hopefully, the present findings can provide new insights into the reactivity of the CH radical in the transformation of TCDD-like dioxins.

## 1. Introduction

As one of the important representative persistent organic pollutants, polychlorinated dibenzo-*p*-dioxins (PCDDs) are a class of environmental pollutants that arise from the uncontrolled combustion process in the presence of chlorine, e.g., forest fires and volcanic activity. They have been studied extensively due to their highly toxicological effects (e.g., hepatotoxicity, immunotoxicity, dermal toxicity, embryotoxicity, and carcinogenicity etc.). Among the family of PCDDs, 2,3,7,8-tetrachlorodibenzo-*p*-dioxin (TCDD) has been recognized as the most toxic dioxin. At present, many studies have been carried out experimentally and theoretically, mainly focusing on the formation [1,2,3,4,5,6,7,8] and transformation or degradation [9,10,11,12,13,14,15,16,17,18,19,20,21,22,23,24,25,26,27] mechanisms of PCDDs. Especially, several methods, such as microbial degradation [9,10,11], photocatalytic decomposition [12,13,14], and high temperature degradation [15], have been proposed to degrade PCDDs. On the other hand, the high stabilities of PCDDs due to their specific structures limit their degradation or transformation in practice. Actually, only a few free radicals such as OH [19,20,21,22] and NO_3_ [22,23] can react with PCDDs. Therefore, it is necessary to find new reactive species which can react with PCDDs so as to provide important clues to the transformation of PCDDs for experiments. 

As a highly reactive radical, methylidyne radical (CH) plays an important role in the fields of combustion, interstellar medium and planetary atmospheric chemistry [28,29]. Not only has it been detected unambiguously in diffuse clouds in the interstellar medium and Titan’s atmosphere [30,31,32], but it also can be prepared via the photolysis of bromoform at room temperature experimentally. Due to the extreme reactivity of CH radical, its reactions with other species have been explored experimentally and theoretically. For example, CH radical can react with most of the saturated and unsaturated hydrocarbons [33,34,35,36,37,38,39,40,41,42,43,44,45], pyrrole [46], acrolein [47], and anthracene (C_14_H_10_) [48] through insertion or addition modes, providing alternative approaches for the syntheses of novel long-chain or ring-expanded species. Generally, these reactions are fast, highly exothermic, and barrierless [49,50], exhibiting a negative temperature dependence on the rate coefficients.

In our previous study [51], the reaction of CH radical with 2,3,7,8-tetrachlorodibenzofuran (TCDF) dioxin was investigated theoretically. It was found that the reaction can proceed preferentially through the insertion of the CH radical into the C-C bond of TCDF except for that of the C atom attached directly to the chlorine atom. Moreover, the main products have been confirmed by the molecular dynamics. Given the similar structure between TCDD and TCDF, we wonder if TCDD can react with the CH radical. If it can, what are the differences between the two types of reactions? To answer these questions, a systematic study on the reaction of the CH radical with TCDD is highly desirable in the case of lacking the relevant experiments.

Therefore, to further acquire a more comprehensive understanding of the reactivity of the CH radical toward dioxins, in the present study, the reaction mechanism between CH radical and TCDD has been systematically explored by employing the DFT method and *ab initio* molecular dynamics simulations. The relevant thermodynamic and kinetic parameters in the possible reaction pathways have been discussed to determine the most favorable pathways of the title reaction. Moreover, the IR and hyperfine coupling constants for the major products have been predicted to provide help for experimental detection and identification of them. In addition, the reactions of the CH radical with the F- and Br-substituted TCDD dioxins have also been investigated to further testify the reactivity of CH radical. Expectedly, the present results can provide new insights into the reaction mechanism between CH radical and TCDD dioxin as well as an alternative approach for the transformation of TCDD-like dioxins.

## 2. Computational Details

Recently, the density functional theory (DFT) method has been widely applied in a variety of systems since it can provide many properties comparable in accuracy to experimental results and higher-level theories at a modest computational cost [36,37,38,39,40,51,52,53,54,55,56,57,58,59,60,61]. In this study, considering the compromise between the computational cost and accuracy as well as to keep the consistency of the results for the optimization and molecular dynamics calculations using the same method, the B3LYP method [62,63] has been employed plus the 6-311++G** basis set, which has also been used to explore the reactivity of CH radical toward TCDF dioxin [51]. Meanwhile, vibrational frequency calculations have been carried out at the same level of theory to identify the nature of the optimized structures. In addition, intrinsic reaction coordinate (IRC) calculations have been done to further confirm the connectivity between the transition states and the related reactants and products [64,65]. All the energy parameters have been corrected by the zero-point vibrational energy (ZPVE) if not noted otherwise.

To further confirm the choice of the B3LYP/6-311++G** level of theory, relevant calculations at the B3LYP/AUG-cc-pVTZ and M06-2X/6-311++G** levels of theory have been performed for the representative reaction pathway, where the former is the single point energy calculations based on the optimized geometries at the B3LYP/6-311++G** level of theory. As mentioned below, the calculated results are well consistent with those at the B3LYP/6-311++G** level of theory. For simplicity, the results at the B3LYP/6-311++G** level of theory have been mainly discussed below if not noted otherwise. 

To characterize the structural features and nature of the interatomic interactions for the initial intermediates and products, topological analyses for the optimized geometries have been performed employing the atoms in molecules (AIM) theory [66]. In AIM analyses, the interatomic interaction is indicated by the presence of a bond critical point (BCP). Similarly, the ring structure is characterized by the presence of a ring critical point (RCP). The topological parameters, such as the Laplacian (∇^2^*ρ*_bcp_) and energy density (*H*_bcp_) including the kinetic energy and potential energy density at the BCP, can be used to predict the interaction types. Moreover, to further clarify the existence of the non-covalent interactions in the selected products, the reduced density gradient (RDG) analyses have been performed based on the optimized structures. In this method, the non-covalent interaction can be intuitively illustrated by the appearance of the corresponding spikes between fragments [67,68]. 

To explore the interconversion among the initial intermediates and the formation processes of the products, *ab initio* molecular dynamics has been performed at the B3LYP/6-311G** level of theory employing the Atom Centered Density Matrix Propagation (ADMP) approach on the basis of the optimized initial intermediates [69,70,71]. Note that ADMP belongs to the extended Lagrangian approach to molecular dynamics using Gaussian basis functions and propagating the density matrix. Fictitious masses for the electronic degrees of freedom are set automatically and can be small enough that thermostats are not required for good energy conservation. Here, the dynamics calculations were performed with a NVT ensemble (constant number of particles, constant volume, and constant temperature) at 298 K and the total simulation time is 1.0 ps with a time stepsize of 1 fs. 

For the isotropic hyperfine coupling constant for a nucleus N, it is calculated according to the following formula: [72]

(1) $$\alpha_{\mathrm{iso}}\left(N\right)=\left(\frac{4\pi }{3}\right){\mathrm{gg}}_{N}\beta \beta_{N}\langle S_{Z}\rangle {}^{-1}\rho \left(N\right)$$

where g is the electronic g-factor; *β* is the Bohr magneton; $g_{N}$ and *β*_N_ are the analogues for nucleus N. *ρ*(N) is the Fermi contact integral calculated in terms of the following formula:

(2) $$\rho \left(N\right)=\underset{\mu \nu }{\sum }P_{\mu \nu }^{\alpha -\beta }\langle \phi_{\mu }\left(r_{\mathrm{kN}}\right)\left|\delta \left(r_{\mathrm{kN}}\right)\right|\phi_{\nu }(r_{\mathrm{kN}})\rangle$$

where $P_{\mu \nu }^{\alpha -\beta }$ is an element of the one-electron spin density matrix and  ϕ designates the atomic basis functions.

All the calculations have been completed by using Gaussian 09 program [73].

## 3. Results and Discussion

**Figure 1:**
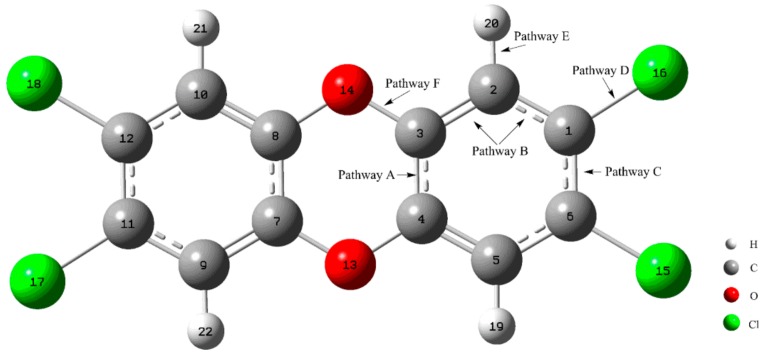
The C-X (X=C, Cl, H, O) bonds of TCDD attacked by CH radical in the studied pathways.

As displayed in Figure 1, CH radical can attack the four different C-X (X=C, Cl, H, O) bonds of TCDD in terms of the symmetry of TCDD. The relevant pathways have been discussed in detail below.

### 3.1. Formation of Initial Intermediates

**Figure 2:**
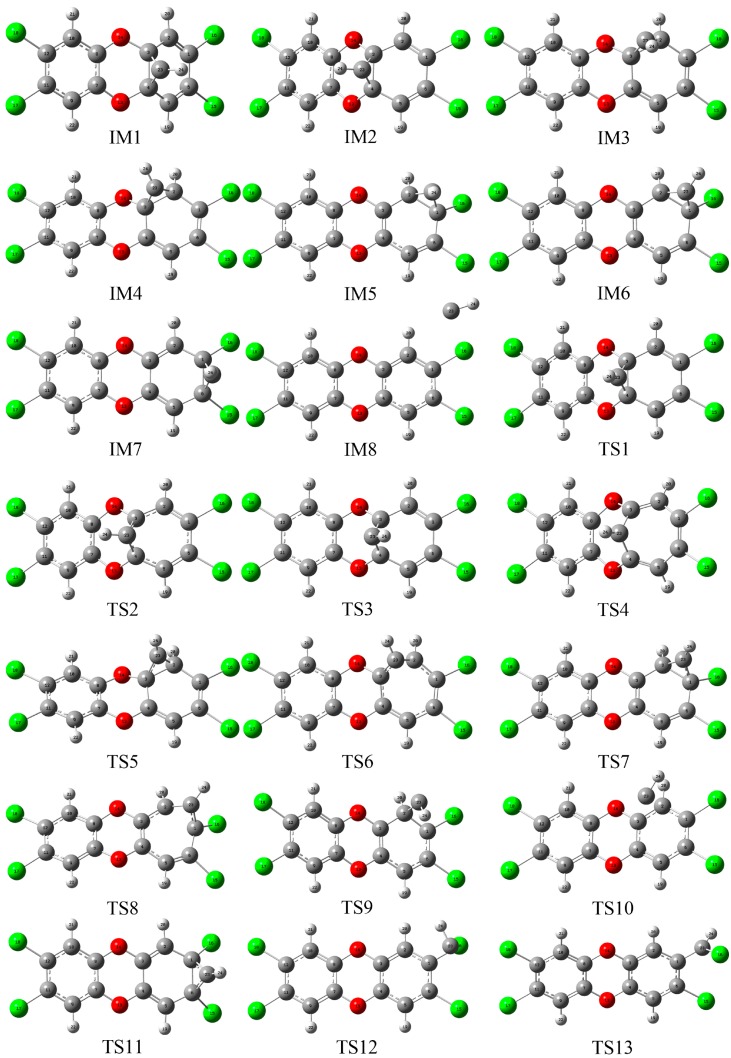

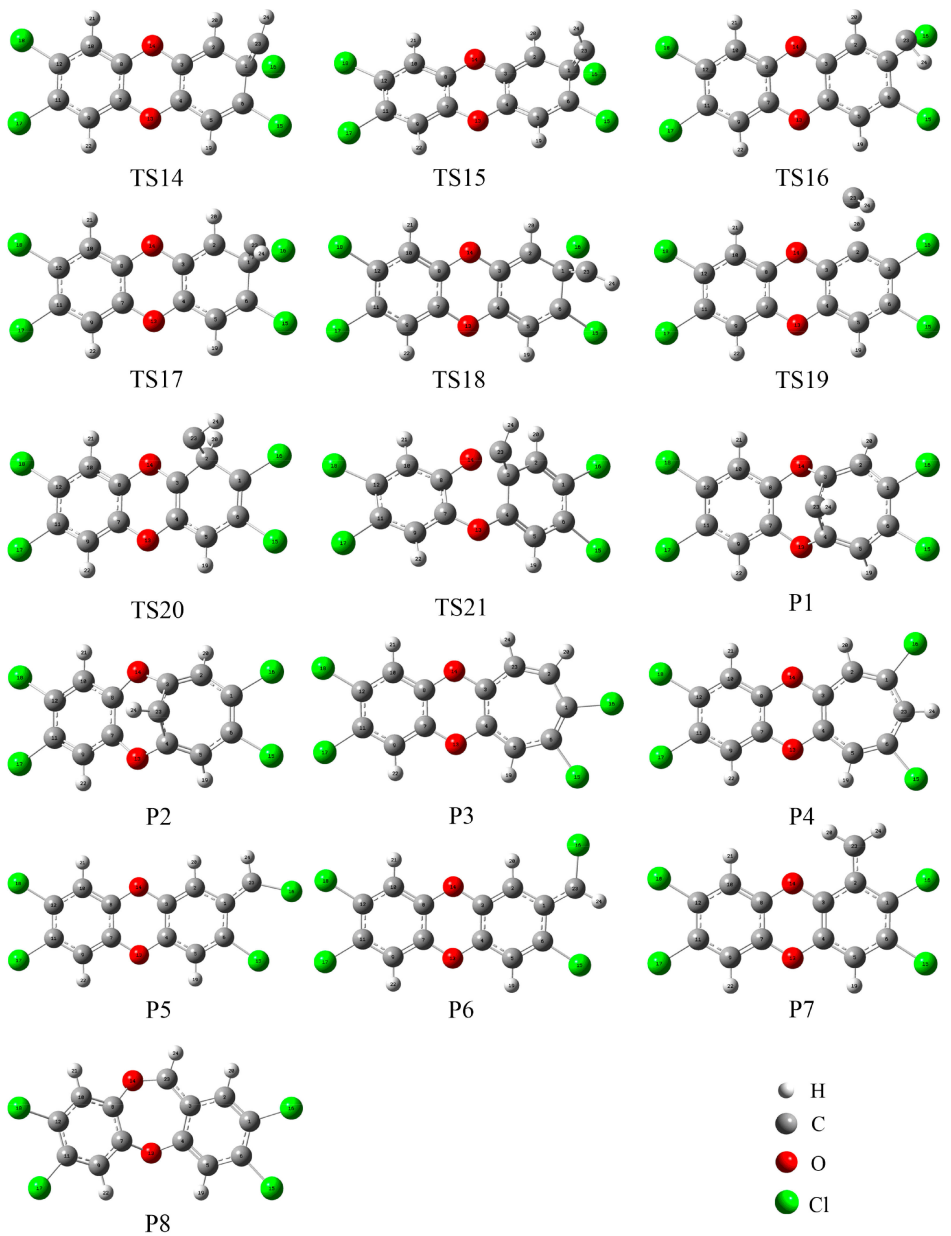
Optimized initial intermediates (IM), transition states (TS), and products in all the reaction pathways. The Cartesian coordinates of them have been given in Supporting Information for reference.

As the first step of the whole reaction, the formation of the initial intermediates (IMs) was explored first. As displayed in Figure 2, eight initial IMs named as IM1-IM8 have been located barrierlessly in the possible pathways, where the corresponding relative energies and thermodynamic data including the enthalpy and Gibbs free energy changes (ΔH and ΔG) in the formation processes have been summarized in Table 1. Obviously, all the IMs have been stabilized by about 42–51 kcal/mol except for IM8. Moreover, the large negative ΔH and ΔG results suggest that the formation processes of IMs are strongly exothermic and spontaneous reactions except for IM8. As for IM8, the relative energy and small negative ΔH and ΔG results suggest that its formation is unfavorable thermodynamically. In addition, as shown in Table 1, the calculated results for IM1 and IM2 at the B3LYP/AUG-cc-pVTZ level of theory are consistent with those at the B3LYP/6-311++G** level of theory.

**Figure 3:**
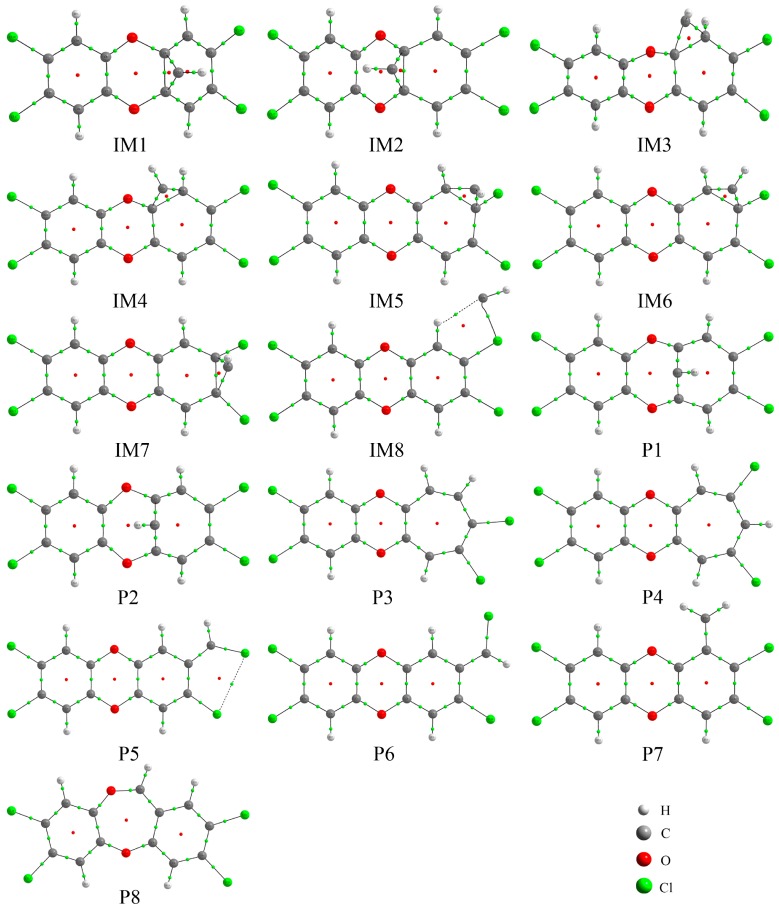
Molecular graphs of the initial intermediates and products, where the BCP and RCP are denoted as small green and red dots, respectively.

As shown in Figure 3, the presence of the RCP suggests that a new three-membered ring structure has been formed between the C atom of the CH radical and two adjacent C atoms of TCDD via cycloaddition mechanism except for IM8, where a five-membered ring has been formed between the CH radical and the H and Cl atoms of TCDD in IM8. Moreover, as presented in Table S1 of the Supporting Information (SI), the calculated negative ∇^2^*ρ*_bcp_ values at the BCP of the C-C bond between CH radical and TCDD suggest that the newly formed C-C bond should be a covalent bond, which is consistent with the above large relative energies, ΔH, and ΔG in the formation processes of them. However, the positive ∇^2^*ρ*_bcp_ values at the two BCPs between CH and TCDD in IM8 suggest the existence of the non-covalent interactions between them. 

**Figure 4:**
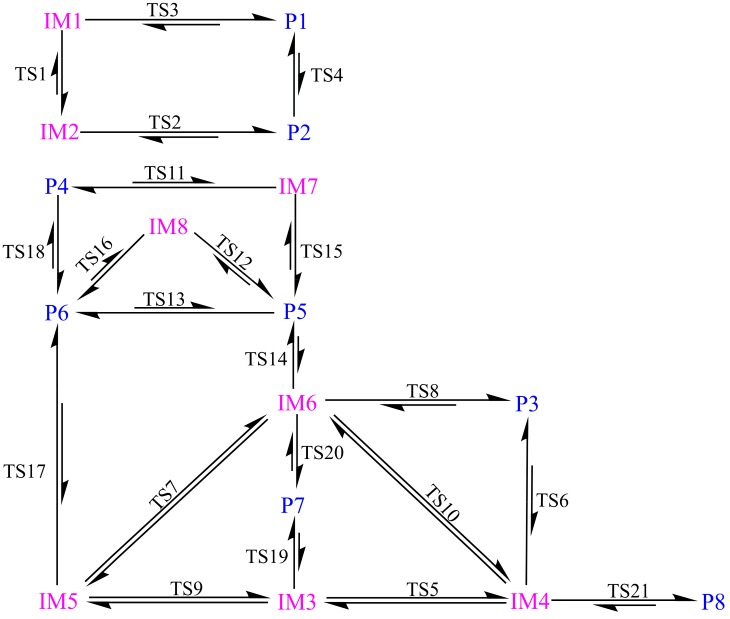
Schematic diagram for the interconversion among initial intermediates and products. The short and two-way arrows denote the high barrier height for the forward reaction and the almost equivalent barrier height for the forward and reverse reaction, respectively.

**Figure 5:**
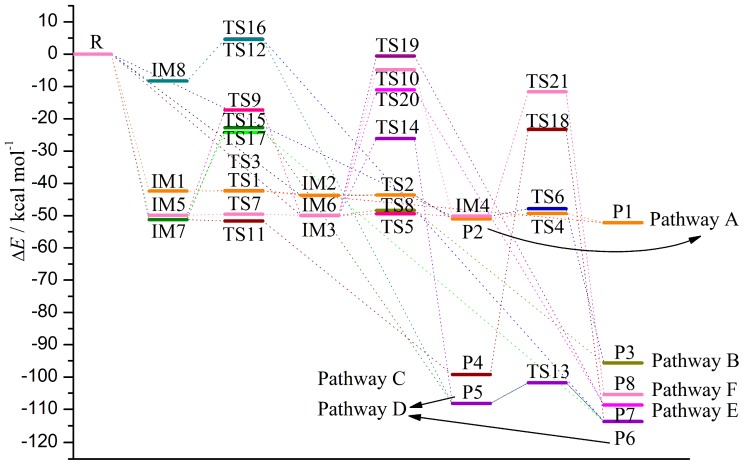
Reaction profiles for the reaction between TCDD and CH radical.

Subsequently, eight products, P1–P8, have been produced arising from the transformation of IMs via different pathways. Twenty-one transition states, TS1–TS21, have been located in the interconversion process among IMs and the formation processes of the products. Here, the relative energies for TSs, forward and reverse barrier heights, and thermodynamic parameters (ΔH and ΔG) in the above processes have been summarized in Table 2. For the sake of simplicity, the relationships among the IMs, TSs, and products have been shown in Figure 4. The reaction profile for the title reaction is displayed in Figure 5. 

### 3.2. Reaction Pathways

#### 3.2.1. Pathway A

As shown in Figure 1 and Figure 2, in this pathway, CH radical attacks the C3-C4 bond of TCDD, resulting in the formation of two intermediates, IM1 and IM2, firstly. Analyses of the geometries of IM1 and IM2 suggest that their differences are only the different orientations of the H atom in the introduced CH radical, i.e., both H atoms lie on the sides of the formed three-membered ring, implying the similar stability of them. As shown in Figure 4 and Figure 5, IM1 and IM2 can interconvert into each other via transition state TS1, i.e., IM1 **⇌** IM2. As expected, the H atom of the introduced CH radical is almost in the plane of the three-membered ring in TS1. The short distance for the H atom transfer from IM1 to TS1 implies the low barrier height. In fact, for this process, the corresponding ΔH and ΔG results are −1.36 (−0.46) and −1.09 (−0.54) kcal/mol, respectively, where the data in parentheses refer to the results at the M06-2X/6-311++G** level of theory. Obviously, this interconversion process is a favorable spontaneous and exothermic reaction thermodynamically. Moreover, the forward and reverse barrier heights are −0.01 (0.69) and 1.31 kcal/mol, where the value in parentheses is the result without including ZPVE corrections. Here, the disappearance of the forward barrier height suggests that it is easy to take place for the conversion from IM1 to IM2 kinetically. In addition, low forward and reverse barrier heights of 0.58 (0.03) and 1.04 (1.38) kcal/mol have been obtained at the M06-2X/6-311++G** (B3LYP/AUG-cc-pVTZ) level of theory.

After then, IM1 and IM2 can be converted into products P1 and P2 via transition states TS3 and TS2, respectively, i.e., IM1→TS3→P1 and IM2→TS2→P2, where both products are characterized by a non-planar seven-membered ring structure. For the two processes, the calculated ΔH (ΔG) results are −10.00 (−9.00) and −7.40 (−6.96) kcal/mol, respectively. Moreover, the corresponding barrier heights are 0.08 and 0.09 kcal/mol, respectively. Therefore, IM1 and IM2 can be transformed into P1 and P2 easily. 

#### 3.2.2. Pathway B

As shown in Figure 1 and Figure 2, in this pathway, CH radical attacks the C1-C2 and C2-C3 bonds of TCDD, resulting in the formation of four intermediates IM3, IM4, IM5, and IM6. As shown in Figure 4 and Figure 5, these four IMs can interconvert into each other via the corresponding transition states TS9, TS7, TS5, and TS10, i.e., IM5 ⇌ IM3, IM5 ⇌ IM6, IM3 ⇌ IM4, and IM6 ⇌ IM4, where the forward (reverse) barrier heights are 32.62 (32.72), 0.33 (0.35), 0.55 (0.72), and 45.08 (45.34) kcal/mol, respectively. Moreover, the corresponding ΔH (ΔG) results are −0.22 (0.31), −0.01 (−0.08), −0.17 (−0.23), and −0.38 (0.15) kcal/mol, respectively. Obviously, it is feasible only for the processes IM5 **⇌** IM6 and IM3 ⇌ IM4 to take place as can be seen from the negative ΔH and ΔG results as well as the low barrier heights for them. Therefore, IM3 and IM5 can easily be converted into IM4 and IM6, respectively. 

Subsequently, IM4 and IM6 can be further converted into P3 via transition states TS6 and TS8, i.e., IM4→TS6→P3 and IM6→TS8→P3, where P3 is characterized by a planar seven-membered ring structure. The barrier heights of the two processes are only 2.28 and 1.51 kcal/mol, respectively. Moreover, both processes are spontaneous and highly exothermic reactions, where the corresponding ΔH (ΔG) results are −45.12 (−46.89) and −45.50 (−46.73) kcal/mol. In addition, considering the easy conversion of IM3 and IM5 into IM4 and IM6 as mentioned above, one can say that P3 should be the major product in this pathway, where the process IM6→TS8→P3 is the most favorable channel to produce P3. 

#### 3.2.3. Pathway C

As shown in Figure 1 and Figure 2, in this pathway, CH radical attacks the C1-C6 bond of TCDD, resulting in the formation of a three-membered ring intermediate IM7. Subsequently, IM7 undergoes transition states TS11 to produce the product P4, i.e., IM7→TS11→P4, where P4 is characterized by a planar seven-membered ring structure. Note that this process is a spontaneous and highly exothermic reaction, where the corresponding ΔH (ΔG) is −47.96 (−48.41) kcal/mol. Moreover, the barrier height of this process is −0.41 (0.21) kcal/mol, where the value in parentheses is the result without considering the ZPVE corrections. Here, the disappearance of the forward barrier height suggests that this reaction can proceed readily. Therefore, P4 should be the major product in this pathway.

As for the reaction of the CH radical with 2,3,7,8-tetrachlorodibenzofuran [51], a similar product has also been obtained by the insertion of the CH radical into the same C-C bond. However, the obtained product is not the major product in the reaction.

#### 3.2.4. Pathway D

As shown in Figure 1 and Figure 2, in this pathway, CH radical attacks the C1-Cl16 bond of TCDD, resulting in the formation of IM8. After then, IM8 can undergo two different transition states to produce P5 and P6, i.e., IM8→TS12→P5 and IM8→TS16→P6, where the corresponding barrier heights of 12.88 and 12.94 kcal/mol are larger than those processes to produce P1–P4. Meanwhile, the two reaction processes are exothermic and spontaneous processes, where the corresponding ΔH (ΔG) results are −100.58 (−99.14) and −106.15 (−104.49) kcal/mol, respectively. Moreover, further IRC analyses suggest that IM5, IM6, and IM7 can also be converted into products P5 and P6 via the following processes, i.e., IM6→TS14→P5, IM7→TS15→P5, and IM5→TS17→P6. However, the relatively high barrier heights ranging from 23.76 to 28.50 kcal/mol suggest that these three processes occur with difficulty. In addition, as the precursors to products P5 and P6, the formation of IM8 is also unfavorable relative to other intermediates thermodynamically. Therefore, P5 and P6 should be the insignificant products in the title reaction. 

#### 3.2.5. Pathway E

As shown in Figure 1 and Figure 2, in this pathway, CH radical attacks the C2-H20 bond of TCDD to produce product P7. However, further IRC analyses suggest that P7 originates from the conversion of IM3 and IM6, i.e., IM3→TS19→P7 and IM6→TS20→P7. The calculated ΔH and ΔG results of −58.56 (−59.03) and −58.77 (−58.64) kcal/mol suggest that the above two processes are feasible thermodynamically since they are spontaneous and exothermic processes. On the contrary, the calculated high barrier heights (49.41 and 38.87 kcal/mol) suggest that those processes are difficult to proceed. Therefore, the possibility of obtaining P7 is small. 

#### 3.2.6. Pathway F

As shown in Figure 1 and Figure 2, in this pathway, CH radical attacks the C3-O14 bond of TCDD, resulting in the formation of the ring-expanded product P8. Further IRC analyses suggest that P8 originates from the conversion of IM4, i.e., IM4→TS21→P8. On the other hand, the calculated barrier height of 38.50 kcal/mol for this process is too high to occur, although it is an exothermic and spontaneous process thermodynamically. Here, the corresponding ΔH and ΔG results are −55.26 and −55.53 kcal/mol, respectively. Compared with the above process IM4→TS6→P3 to produce P3 with a low barrier height of 2.28 kcal/mol, the P8 is difficult to obtain in practice. 

Additionally, the hydrogen abstraction channel has been explored. As a result, it was found that the calculated ΔH (ΔG) results are 25.00 and 22.74 kcal/mol for the whole reaction process. Therefore, these large positive results for the thermodynamic parameters suggest that the hydrogen abstraction channel here is infeasible in practice.

### 3.3. Relative Stability and Interconversion for Products

#### 3.3.1. Relative Stability

As shown in Figure S1 of the SI, we have calculated the relative energies for all the products relative to TCDD and CH radical. Clearly, the orders of the relative stability for these products are as follows: P6 > P7 > P5 > P8 > P4 > P3 > P1 > P2. To explore the relationship between the stability of the product and its single electron distribution, the spin density distributions for these products have been investigated.

**Figure 6:**
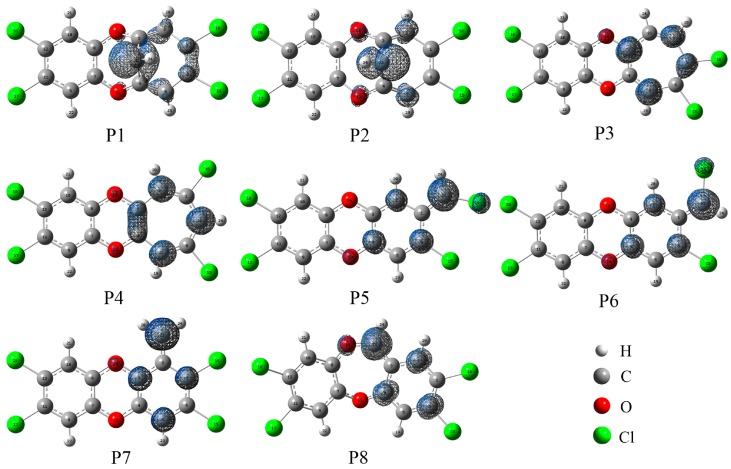
Spin density maps for the products. The isodensity contours are 0.005 electron/bohr^3^.

**Figure 7:**
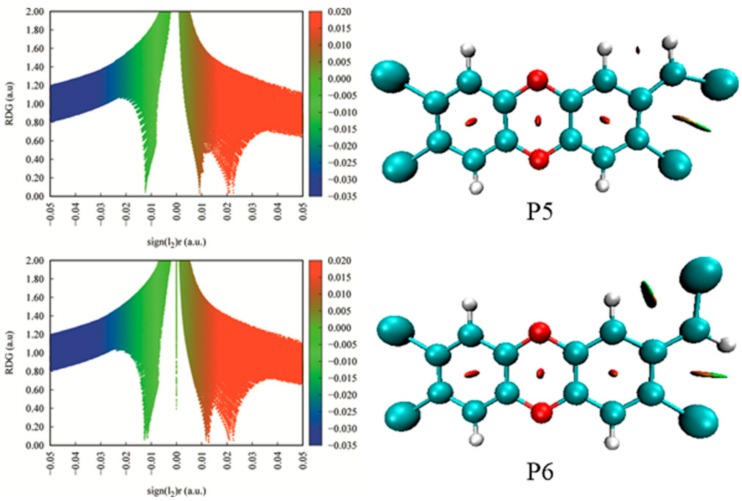
RDG analyses for products P5 and P6.

As displayed in Figure 6, the distributions of the single electron are different depending on the specific insertion mode for CH radical. Overall, these products can be classified into three groups in terms of the degrees of the single electron delocalization. In the first group, i.e., P5, P6, P7, and P8, the single electron is uniformly distributed on the six-membered ring and the C atom of the introduced CH radical. Moreover, as displayed in Figure S1 of the SI, four products have similar stabilities. Compared with P5, the most stable product, P6, should be attributed to the existence of the intramolecular H-bonds. As shown in Figure 7, the intramolecular H-bond H20···Cl16 and H24···Cl15 in P6 can be verified by the presence of the corresponding spikes in RDG analyses. On the contrary, the repulsion interactions H20···H24 and Cl15···Cl16 can be observed in P5. In the second group including P3 and P4, the single electron is mainly distributed on the seven-membered ring. Obviously, the large tension of the seven-membered ring in the product may reduce its stability relative to that of the six-membered ring product. In the third group including P1 and P2, the single electron distributions of them are similar to each other and are mainly distributed on the C atom of the introduced CH radical. Especially, the above classifications can also be applied for the relative stabilities among these products. Therefore, one can say that the ability of the product is mainly governed by the single electron distribution, i.e., the more delocalization for the single electron, the more stable for the product. Similarly, this phenomenon has also been observed in the reaction of TCDF with CH radical [51].

#### 3.3.2. Interconversion among Products

As displayed in Figure 4 and Table 2, the interconversion among products can be observed. For example, for the three processes, i.e., (1) P2→TS4→P1, (2) P4→TS18→P6, and (3) P5→TS13→P6, the ΔH (ΔG) results are −1.24 (−0.95), −14.66 (−14.06), and −5.57 (−5.35) kcal/mol, respectively. Therefore, the above processes should be exothermic and spontaneous reactions thermodynamically. Meanwhile, the corresponding barrier heights for them are 1.71, 75.91, and 6.42 kcal/mol, respectively. Obviously, it is feasible for the processes (1) and (3) because of their low barrier heights kinetically.

As given in Table 2, for the formation processes of P1–P4, the low barrier heights less than 3 kcal/mol and large negative ΔH and ΔG results suggest that products P1–P4 are easy to be produced. On the contrary, for the products P7 and P8, the high barrier heights ranging from 38.50 to 49.41 kcal/mol suggest that it is difficult to produce P7 and P8 although the formations of them are favorable thermodynamically. As for the formation of products P5 and P6, the corresponding barrier heights range from 12.88 to 28.50 kcal/mol, suggesting that they have no competition with the products P1–P4. Meanwhile, as the precursor to P5 and P6, the formation of IM8 is unfavorable compared with the other initial intermediates. In addition, as mentioned above, P2 is easy to convert into P1. Therefore, P1, P3 and P4 should be the major products in the title reaction, which can be further confirmed by the molecular dynamics simulations below.

### 3.4. Molecular Dynamics Simulations

To further verify the major products in the title reaction and their dynamic stability, *ab initio* molecular dynamics calculations have been carried out based on the optimized geometries of IMs.

**Figure 8:**
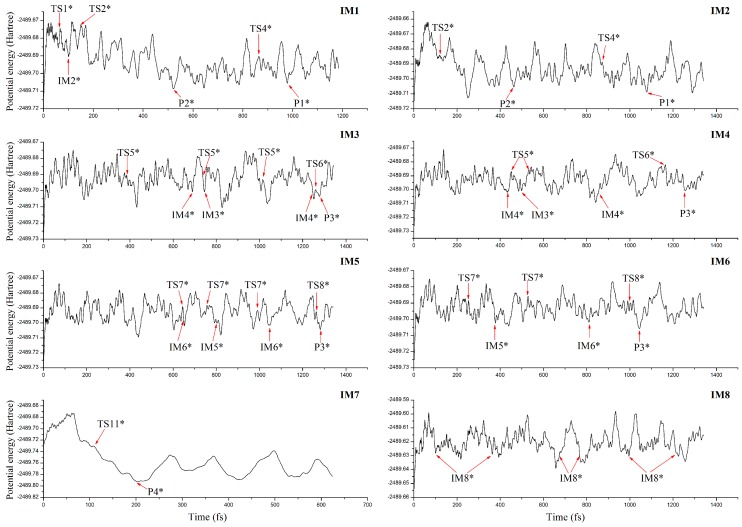
Evolution of the potential energy of the initial intermediates as a function of simulation time, where the asterisk denotes the selected geometry analogous to the optimized intermediate, transition state, and product.

As displayed in Figure 8, the evolution of the initial intermediates along with the simulation time can be clarified, where all the geometries marked in Figure 8 can be further optimized into the nearest species. For IM1, it can be transformed into P1 eventually, which is consistent with the above optimization results. Moreover, the following interconversion processes, IM1→TS1→IM2, IM2→TS2→P2, P2→TS4→P1, can also be reproduced in the dynamics simulations. Similarly, IM2 can also be transformed into P1. As for IM3, IM4, IM5, and IM6, they have been transformed into P3 at different time scales. For IM7, it can be transformed into P4 via TS11, which is well consistent with the low barrier height mentioned above. On the other hand, as for IM8, no corresponding products have been observed due to the high barrier heights mentioned above. Given the fact that it is unfavorable to form IM8 thermodynamically, the products P1, P3 and P4 should be the major products for the title reaction.

### 3.5. Analyses of IR Spectra and Hyperfine Coupling Constants

To provide helpful information concerning the experimental detection and identification for the products employing IR and electron spin resonance (ESR) techniques, the IR and hyperfine coupling constants (hfcc’s) of the major products mentioned above have been discussed.

#### 3.5.1. IR Spectra

**Figure 9:**
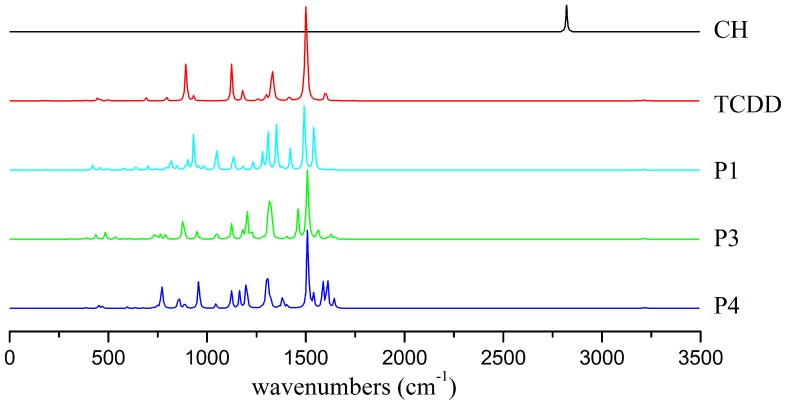
IR spectra of the monomers and major products.

As displayed in Figure 9, the characteristic peaks of CH radical and TCDD appear at 2821 and 1501 cm^−1^, which are assigned to the stretching vibration of the C-H bond in CH radical and the rocking mode of the C-H bond in the ring of TCDD, respectively. However, for the C-H vibration of CH radical, its adsorption peak disappears upon formation of products. As for the rocking mode of the C-H bond in TCDD, it is still the characteristic peak in the products, which appears at 1493 (P1), 1508 (P3), and 1510 cm^−1^ (P4), respectively. Moreover, the distinct adsorption peaks for each product can be used to identify them. For example, another strong adsorption peak appears at 1542 cm^−1^ in P1, corresponding to the rocking mode of the C-H bond and the stretching vibration of the C-C bond in the seven-membered ring. As for P3, a strong adsorption peak can be observed at 1460 cm^−1^, mainly corresponding to the rocking mode of the C-H bond in the seven-membered ring. Two adjacent peaks appear at 1586 and 1609 cm^−1^ in P4, which mainly correspond to the stretching vibration of the C-C bond of the ring. Obviously, these different characteristics of IR spectra for products can provide help in the identification of them experimentally.

#### 3.5.2. Hyperfine Coupling Constants

At present, ESR technique is an efficient method to characterize radical systems due to the high reactivity of radicals. To provide useful information for the relevant ESR experiments, we have calculated the hfcc’s of the major products.

As presented in Table 3, the different hfcc results in magnitudes and signs provide convenience for the identification of these products. For example, for the ^13^C1 atom, its hfcc result is 0.1, 2.4 and −11.0 G for P1, P2 and P3, respectively. As for the ^13^C23 atom of the introduced CH radical, its hfcc is 60.5, 4.2 and 10.9 G in P1, P3 and P4, respectively. 

### 3.6. Substitution Effects

To further clarify the reactivity of the CH radical towards other TCDD derivatives, the reactions of CH radical with the F- and Br-substituted TCDDs have been explored on the basis of the relevant pathways to produce the major products P1, P3 and P4, as mentioned above. As displayed in Figure S2 of the SI, the relevant intermediates, transition states, and products involved in the reactions have been located, which are similar to these of the geometries before substitution. Similarly, as presented in Table 1 and Table 2, all the negative ΔH and ΔG results and low barrier heights less than 3 kcal/mol in these reactions suggest that all these reactions can proceed readily thermodynamically and kinetically. For comparison, the calculated Δ*E*_rela_, ΔH, and ΔG results decrease from F- to Br-substitution for the formation of IM1 and IM2. On the contrary, the opposite is true for the formation of IM4, IM6, and IM7. As for the calculated barrier heights, no obvious rules have been observed for the substitution. Therefore, CH radical can react with TCDD and its derivatives easily, providing an alternative approach for the transformation of TCDD dioxins. Certainly, further experiments are required to verify this point. 

## 4. Conclusions

In the present study, the reactions of the CH radical with TCDD and its F- and Br-substituted derivatives have been systematically explored by using the DFT and molecular dynamics simulations. It was found that CH radical can react with TCDD dioxins easily thermodynamically and kinetically. An initial intermediate can be formed barrierlessly first. After, the initial intermediate can be further transformed into the product. Among the possible reaction pathways, insertion of the CH radical into the C-C bond of TCDD is the most favorable channel. Moreover, the major products in the reaction have been further confirmed by the molecular dynamics calculations, which are characterized by a seven-membered ring structure. The distinct features of the IR spectra and the hfcc of the three major products facilitate their detection and identification experimentally. In addition, the feasibility of the title reaction has been further testified by the reactions involving the F- and Br-substituted TCDDs. Expectedly, the present findings provide new insights into the reactivity of the CH radical in the transformation of TCDD-like dioxins. On the other hand, note that the realistic title reaction is more complex than the proposed pathways mentioned above and the major products are not necessarily the ones with the lowest barriers or the most exothermicity. Therefore, further experiments are highly desirable to verify the present results.

## Figures and Tables

**Table 1:** Calculated relative energies (Δ*E*_rela_) and the enthalpy and Gibbs free energy changes (ΔH and ΔG) in the formation process of initial intermediates *^a.^*

Intermediates	Δ*E*_rela_	Δ*H*	Δ*G*
IM1	−42.41 (−*44.30/*−*42.17*) [−41.37]	−43.70 (−*45.77/*−*43.48*) [−42.67]	−34.07 (−*35.32/*−*33.95*) [−33.03]
IM2	−43.72 (−*45.54/*−*43.49*) [−42.72]	−45.06 (−*46.93/*−*44.85*) [−44.06]	−35.16 (−*36.63/*−*34.87*) [−34.16]
IM3	−49.98	−51.37	−41.21
IM4	−50.16 (−*48.74/*−*50.43*)	−51.54 (−*50.13/*−*51.82*)	−41.44 (−*39.76/*−*41.64*)
IM5	−49.88	−51.15	−41.52
IM6	−49.90 (−*49.87/*−*51.03*)	−51.15 (−*51.17/*−*52.28*)	−41.59 (−*41.31/*−*42.68*)
IM7	−51.26 (−*50.22/*−*53.89*)	−52.36 (−*51.40/*−*54.81*)	−43.06 (−*41.76/*−*46.16*)
IM8	−8.30	−8.83	−1.03

*^a^* All the units are in kcal/mol. The italic data before and after the slash are the F- and Br-substituted results, respectively. The origin of the relative energy, enthalpy, and Gibbs free energy is the sum of the isolated reactants, which are −2489.518429 (−*1048.089817/*−*10945.19916*), −2489.499349 (−*1048.072221/*−*10945.17888*), and −2489.577886 (−*1048.146274/*−*10945.26285*) Hartree for the Cl-(F-/Br-) substituted case, respectively. The data in brackets are the results at the B3LYP/AUG-cc-pVTZ level of theory considering the corresponding thermodynamic corrections at the B3LYP/6-311++G** level of theory.

**Table 2:** Calculated relative energies for the transition state relative to the separated reactants, barrier heights, and the enthalpy and Gibbs free energy changes in the interconversion processes *^a^*.

Reactions	TSs	Δ*E*_rela_	Δ*E*_for_*	Δ*E*_rev_*	Δ*H*	Δ*G*
IM1 ⇌ IM2	TS1	−42.41	−0.01 (0.69)	1.31	−1.36	−1.09
IM2 ⇌ P2	TS2	−43.63	0.09	7.39	−7.40	−6.96
		−*45.36/*−*43.42*	*0.18/ 0.07*	*6.82/7.77*	−*6.77/*−*7.79*	−*6.36/*−*7.39*
IM1 ⇌ P1	TS3	−42.33	0.08	9.86	−10.00	−9.00
		−*42.08/*−*41.99*	*2.22/0.19*	*13.15/10.01*	−*11.06/*−*10.02*	−*10.49/*−*8.88*
P2 ⇌ P1	TS4	−49.30	1.71	2.89	−1.24	−0.95
		−*50.28/*−*49.51*	*1.90/1.68*	*4.95/2.49*	−*3.13/*−*0.86*	−*2.82/*−*0.56*
IM3 ⇌ IM4	TS5	−49.44	0.55	0.72	−0.17	−0.23
IM4 ⇌ P3	TS6	−47.89	2.28	47.80	−45.12	−46.89
		−*47.04/*−*48.30*	*1.70/2.14*	*51.71/46.37*	−*49.49/*−*43.91*	−*51.77/*−*45.60*
IM5 ⇌ IM6	TS7	−49.55	0.33	0.35	−0.01	−0.08
IM6 ⇌P3	TS8	−48.39	1.51	47.29	−45.50	−46.73
		−*48.70/*−*49.55*	*1.17/1.48*	*50.04/45.12*	−*48.45/*−*43.44*	−*50.22/*−*44.55*
IM5 ⇌ IM3	TS9	−17.27	32.62	32.72	−0.22	0.31
IM6 ⇌ IM4	TS10	−4.82	45.08	45.34	−0.38	0.15
IM7 ⇌ P4	TS11	−51.67	−0.41 (0.21)	47.56	−47.96	−48.41
		−*50.57/*−*54.25*	−*0.35 (0.35)/−0.36 (0.00)*	*54.19/44.89*	−*54.50/*−*45.36*	−*55.27/*−*45.62*
IM8 ⇌ P5	TS12	4.58	12.88	112.79	−100.58	−99.14
P5 ⇌ P6	TS13	−101.79	6.42	11.94	−5.57	−5.35
IM6 ⇌ P5	TS14	−26.14	23.76	82.06	−58.26	−58.58
IM7 ⇌ P5	TS15	−22.76	28.50	85.44	−57.06	−57.11
IM8 ⇌ P6	TS16	4.64	12.94	118.36	−106.15	−104.49
IM5 ⇌ P6	TS17	−24.27	25.61	89.46	−63.83	−64.00
P4 ⇌ P6	TS18	−23.31	75.91	90.41	−14.66	−14.06
IM3 ⇌ P7	TS19	−0.58	49.41	108.12	−58.56	−59.03
IM6 ⇌ P7	TS20	−11.03	38.87	97.67	−58.77	−58.64
IM4⇌ P8	TS21	−11.67	38.50	93.79	−55.26	−55.53

*^a^* All the units are in kcal/mol. The direction from left to right is defined as the forward reaction, where Δ*E*_for_* and Δ*E*_rev_* denote the forward and reverse barrier heights, respectively. The data in parentheses denote the results before including ZPVE corrections. The italic data before and after the slash are the F- and Br-substituted results, respectively. The origin of the relative energy is the sum of the isolated reactants, which is −2489.518429 (−*1048.089817/*−*10945.19916*) Hartree for the Cl-(F-/Br-) substituted case.

**Table 3:** Calculated hyperfine coupling constants for the atoms in P1, P3, and P4 *^a^*.

Atomic Labels	Atoms	P1	P3	P4
1	^13^C	0.1	2.4	−11.0
2	^13^C	6.2	−8.4	6.4
3	^13^C	−8.1	12.5	−2.7
4	^13^C	−8.1	−12.7	−2.5
5	^13^C	6.2	10.0	6.2
6	^13^C	0.1	−7.0	−10.9
7	^13^C	−0.1	0.4	−0.3
8	^13^C	−0.1	−0.7	−0.3
9	^13^C	0.2	−0.5	−0.1
10	^13^C	0.2	0.2	−0.1
11	^13^C	0.2	0.7	0.0
12	^13^C	0.2	−0.7	0.0
13	^17^O	−1.0	0.2	−0.1
14	^17^O	−1.0	−0.4	−0.1
15	^35^Cl	0.2	−0.1	−0.1
16	^35^Cl	0.2	0.1	−0.1
17	^35^Cl	0.0	0.0	0.0
18	^35^Cl	0.0	0.0	0.0
19	^1^H	−0.9	−9.8	−7.7
20	^1^H	−0.9	1.1	−7.8
21	^1^H	0.1	−0.3	0.0
22	^1^H	0.1	0.2	0.0
23	^13^C	60.5	4.2	10.9
24	^1^H	−12.0	−6.3	−10.1

*^a^* All the units are in Gauss.

## Supplementary Materials

Supplementary Materials are available online. Figure S1: The schematic maps for the relative energy of products relative to TCDD and CH radical. Figure S2: Optimized IMs, TSs, and products in the reactions of F- and Br-substituted TCDDs with CH radical. Table S1: Topological parameters for the selected BCPs in the initial intermediates and products. Table S2: Cartesian coordinates of the species in the possible pathways.
