# Supplementary material for: Theoretical Investigations on the Reactivity of Methylidyne Radical toward 2,3,7,8-Tetrachlorodibenzo-p-Dioxin: A DFT and Molecular Dynamics Study
Source: Molecules. 2018 Oct 18;23(10):2685. doi: 10.3390/molecules23102685 (PMC6222546; doi:10.3390/molecules23102685)
Supplement: Supplementary file 1 [file molecules-23-02685-s001.pdf]

Theoretical investigations on the reactivity of methylidyne radical toward 2,3,7,8-tetrachlorodibenzo-*p*-dioxin: A DFT and molecular dynamics study

Weihua Wang*, Wenling Feng, Wenliang Wang, Ping Li*
Key Laboratory of Life-Organic Analysis, School of Chemistry and Chemical Engineering, Qufu Normal University, Qufu, 273165, P. R. China

E-mails: wwh78@163.com (Weihua Wang) and lignip@163.com (Ping Li)

Supporting Informaiton

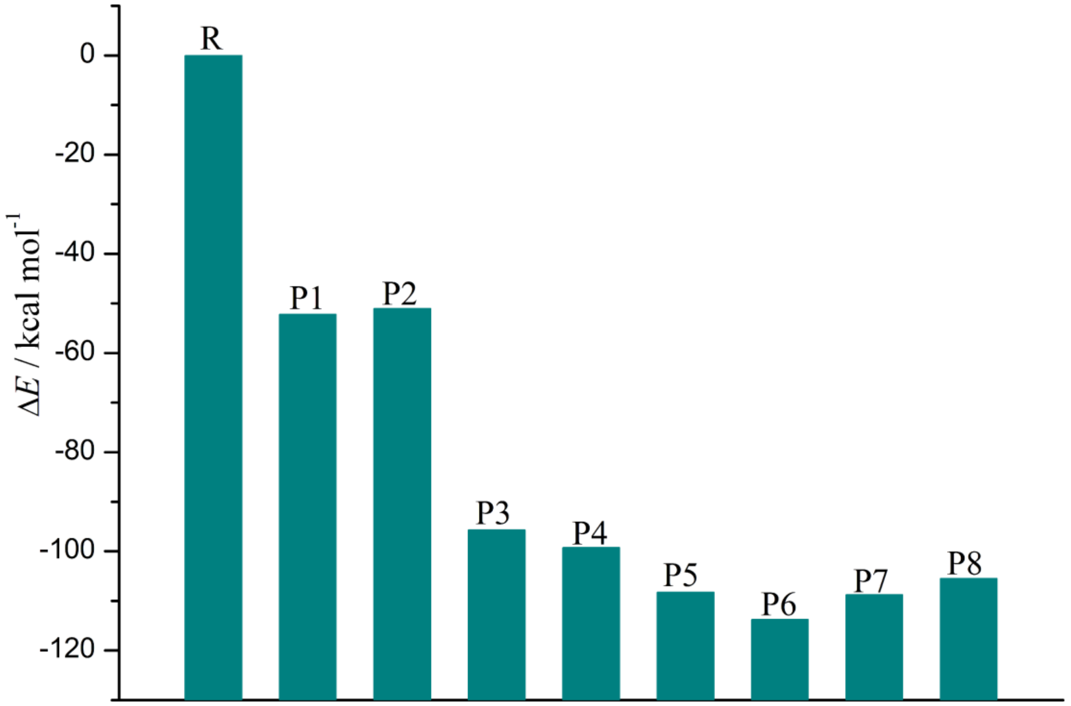

Figure S1. The schematic maps for the relative energy of products relative to TCDD and CH radical.

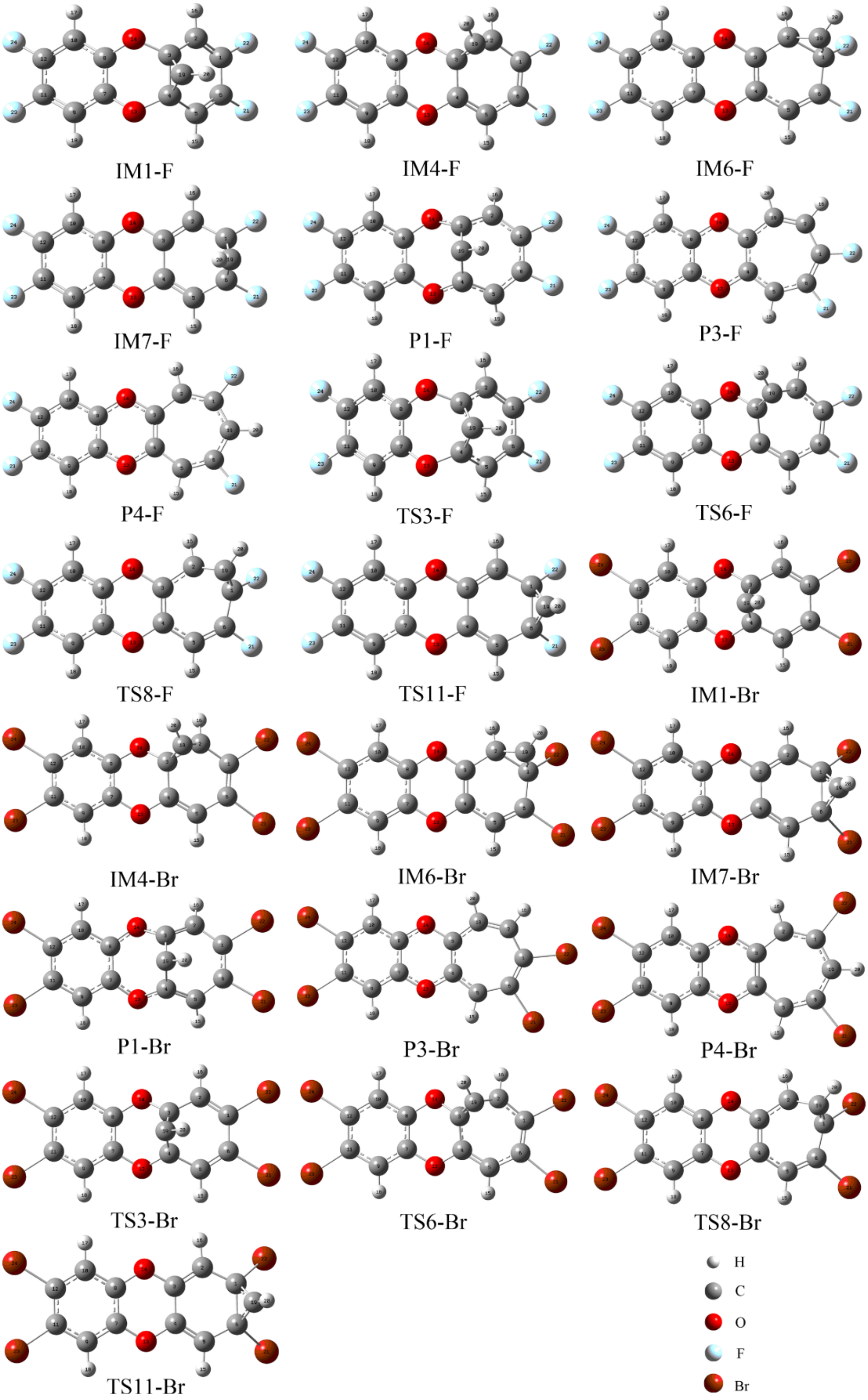

Figure S2. Optimized IMs, TSs, and products in the reactions of F- and Br-substituted TCDDs with CH radical.

Table S1. Topological parameters for the selected BCPs in the initial intermediates and products[a]

Species	BCP	ρ_{bcp}	$\nabla^2\rho_{bcp}$	V_{bcp}	G_{bcp}	H_{bcp}
IM1	C3 - C23	0.2574	-0.5188	-0.3227	0.0965	-0.2262
	C4 - C23	0.2574	-0.5188	-0.3227	0.0965	-0.2262
IM2	C3 - C23	0.2609	-0.5438	-0.3331	0.0986	-0.2345
	C4 - C23	0.2610	-0.5443	-0.3333	0.0986	-0.2347
IM3	C2 - C23	0.2509	-0.4804	-0.3144	0.0971	-0.2172
	C3 - C23	0.2586	-0.5181	-0.3283	0.0994	-0.2289
IM4	C2 - C23	0.2457	-0.4551	-0.3050	0.0956	-0.2094
	C3 - C23	0.2632	-0.5499	-0.3382	0.1004	-0.2378
IM5	C1 - C23	0.2647	-0.5555	-0.3415	0.1013	-0.2402
	C2 - C23	0.2531	-0.4960	-0.3176	0.0968	-0.2208
IM6	C1 - C23	0.2670	-0.5720	-0.3468	0.1019	-0.2449
	C2 - C23	0.2487	-0.4741	-0.3098	0.0956	-0.2142
IM7	C1 - C23	0.2676	-0.5698	-0.3455	0.1015	-0.2440
	C6 - C23	0.2676	-0.5698	-0.3455	0.1015	-0.2440
IM8	Cl16 - C23	0.0891	0.0632	-0.0804	0.0481	-0.0323
	H20 - C23	0.0173	0.0536	-0.0094	0.0114	0.0020
P1	C3 - C23	0.2826	-0.7253	-0.3575	0.0881	-0.2694
	C4 - C23	0.2826	-0.7253	-0.3575	0.0881	-0.2694
P2	C3 - C23	0.2866	-0.7515	-0.3605	0.0863	-0.2742
	C4 - C23	0.2866	-0.7514	-0.3605	0.0863	-0.2742
P3	C1 - C23	0.2773	-0.7127	-0.3311	0.0765	-0.2546
	C2 - C23	0.3230	-0.9207	-0.4662	0.1180	-0.3482
P4	C1 - C23	0.2987	-0.8097	-0.3953	0.0964	-0.2988
	C6 - C23	0.2984	-0.8079	-0.3941	0.0961	-0.2981
P5	C1 - C23	0.2964	-0.7828	-0.3850	0.0947	-0.2904
	Cl16 - C23	0.2044	-0.3032	-0.2222	0.0732	-0.1490
P6	C1 - C23	0.3013	-0.8106	-0.3963	0.0968	-0.2995
	Cl16 - C23	0.2017	-0.2974	-0.2189	0.0723	-0.1466
P7	C2 - C23	0.3037	-0.8288	-0.4082	0.1005	-0.3077
P8	C3 - C23	0.3019	-0.8162	-0.3986	0.0973	-0.3013
	O14 - C23	0.2756	-0.1085	-0.7453	0.3591	-0.3862

[a] Atomic numbering refers to Figure 2. The ρ_{bcp}, $\nabla^2\rho_{bcp}$, V_{bcp}, G_{bcp}, and H_{bcp} is electron density, the Laplacian of the electron density, potential energy density, kinetic energy density, and energy density at the BCP, respectively.

Table S2. Cartesian coordinates of the species in the possible pathways

Species	x	y	z	Species	x	y	z
**TCDD**				**CH**			
C	0.00000000	3.57881500	0.69850100	C	0.00000000	0.00000000	0.16091000
C	0.00000000	2.36878800	1.39107000	H	0.00000000	0.00000000	-0.96545800
C	0.00000000	1.16979600	0.69781000				
C	0.00000000	1.16979600	-0.69781000				
C	0.00000000	2.36878800	-1.39107000				
C	0.00000000	3.57881500	-0.69850100				
C	0.00000000	-1.16979600	-0.69781000				
C	0.00000000	-1.16979600	0.69781000				
C	0.00000000	-2.36878800	-1.39107000				
C	0.00000000	-2.36878800	1.39107000				
C	0.00000000	-3.57881500	-0.69850100				
C	0.00000000	-3.57881500	0.69850100				
O	0.00000000	0.00000000	-1.42570200				
O	0.00000000	0.00000000	1.42570200				
Cl	0.00000000	5.06339400	-1.61505000				
Cl	0.00000000	5.06339400	1.61505000				
Cl	0.00000000	-5.06339400	-1.61505000				
Cl	0.00000000	-5.06339400	1.61505000				
H	0.00000000	2.35436000	-2.47284400				
H	0.00000000	2.35436000	2.47284400				
H	0.00000000	-2.35436000	2.47284400				
H	0.00000000	-2.35436000	-2.47284400				
**IM1**				**IM2**			
C	3.47752700	0.73466600	-0.04654300	C	-3.44439400	-0.73052800	-0.07230300
C	2.35601700	1.44466700	0.18338000	C	-2.34608900	-1.43564500	0.25733100
C	1.11817500	0.78094300	0.60326700	C	-1.14733100	-0.79943100	0.80694800
C	1.11817300	-0.78094700	0.60325900	C	-1.14705800	0.79936800	0.80736700
C	2.35602000	-1.44466700	0.18338100	C	-2.34599100	1.43562800	0.25772800
C	3.47753000	-0.73466300	-0.04653700	C	-3.44420800	0.73060900	-0.07232700
C	-1.19117800	-0.70015300	0.08176800	C	1.16284400	0.70095900	0.25121600
C	-1.19117900	0.70015100	0.08177700	C	1.16276200	-0.70103900	0.25095600
C	-2.39451400	-1.38824300	-0.01540700	C	2.35708600	1.38588900	0.05162600
C	-2.39451500	1.38824200	-0.01538800	C	2.35695500	-1.38594600	0.05089000
C	-3.59770700	-0.69917800	-0.12635700	C	3.54591500	0.69953900	-0.16272200
C	-3.59770800	0.69917800	-0.12634700	C	3.54582600	-0.69958100	-0.16316600
O	-0.03843200	-1.44722500	0.12836500	O	0.03532300	1.46904900	0.42351000
O	-0.03843200	1.44722400	0.12838100	O	0.03544300	-1.46919200	0.42398900
Cl	4.94822500	-1.58573200	-0.45491600	Cl	-4.86304500	1.58824600	-0.62837600
Cl	4.94821400	1.58573700	-0.45494900	Cl	-4.86377200	-1.58794000	-0.62708700
Cl	-5.07863200	-1.61630400	-0.25548500	Cl	5.00910600	1.61682900	-0.42049200
Cl	-5.07863300	1.61630400	-0.25546200	Cl	5.00888300	-1.61687600	-0.42165800

H	2.33597700	-2.51964400	0.05323900	H	-2.30265000	2.50728400	0.10428900
H	2.33596400	2.51964200	0.05322400	H	-2.30312400	-2.50740200	0.10449200
H	-2.38027500	2.47000300	-0.01424600	H	2.34184400	-2.46775100	0.05569400
H	-2.38027400	-2.47000400	-0.01427900	H	2.34203700	2.46769100	0.05690700
C	1.05144100	-0.00000700	1.84756700	C	-1.13006700	-0.00021800	2.03210900
H	1.60908300	-0.00001400	2.77498300	H	-0.35166100	-0.00070100	2.78414100
**IM3**				**IM4**			
C	-3.59944700	-0.60565600	0.06030100	C	-3.60175600	-0.58956700	0.06128100
C	-2.37237700	-1.40186400	0.31577100	C	-2.39778900	-1.40395600	0.34716400
C	-1.01958800	-0.66614300	0.26331800	C	-1.03020400	-0.66959200	0.32755800
C	-1.09115400	0.78820500	0.04768300	C	-1.09814500	0.79054600	0.11299300
C	-2.26236900	1.44664000	-0.05184900	C	-2.25915100	1.45582000	-0.01449200
C	-3.52821400	0.73585500	-0.06586500	C	-3.52442500	0.75154300	-0.06178500
C	1.25755000	0.73752500	-0.03165300	C	1.25273000	0.73819700	0.02761600
C	1.24317100	-0.64475200	-0.23933700	C	1.23678400	-0.64074500	-0.20011500
C	2.46000300	1.41257800	0.11572700	C	2.45814600	1.41269000	0.15713700
C	2.43939900	-1.34518500	-0.30041500	C	2.43273300	-1.33736900	-0.30480500
C	3.66122900	0.71216700	0.04480900	C	3.65829400	0.71642100	0.04612700
C	3.65099700	-0.67085000	-0.16874000	C	3.64567000	-0.66306100	-0.19168200
O	0.09291500	1.47296200	-0.01139900	O	0.09048900	1.47081600	0.08586600
O	0.05413800	-1.30460100	-0.41996000	O	0.04709000	-1.30655000	-0.34910300
Cl	-4.95015400	1.72152800	-0.31200200	Cl	-4.93571400	1.73972400	-0.35202400
Cl	-5.07647100	-1.51168900	-0.09594500	Cl	-5.07910600	-1.48749500	-0.15265600
Cl	5.15442600	1.59876500	0.22785800	Cl	5.15375100	1.60253800	0.20803300
Cl	5.13194100	-1.59040500	-0.26752400	Cl	5.12529100	-1.57719900	-0.34348300
H	-2.26795200	2.52303600	-0.15870500	H	-2.25502300	2.53267600	-0.11572200
H	-2.36239400	-2.39670800	-0.12847900	H	-2.41576600	-2.40022000	-0.09151900
H	2.41927500	-2.41473300	-0.46142900	H	2.41074200	-2.40407000	-0.48371000
H	2.45500000	2.48268800	0.27390600	H	2.45470700	2.48005900	0.33287400
C	-1.53053200	-1.23129400	1.51381600	C	-1.54896900	-1.26208600	1.55367200
H	-1.68797800	-0.85393500	2.51361300	H	-1.13058100	-2.03426400	2.18215400
**IM5**				**IM6**			
C	-3.59133200	-0.65249900	0.14688100	C	-3.58894800	-0.64726900	0.14118000
C	-2.25176800	-1.44176300	0.36740900	C	-2.24793100	-1.43376900	0.40229600
C	-1.00403800	-0.66397600	0.33111600	C	-1.00778100	-0.64822200	0.37343900
C	-1.02152300	0.67752200	0.25190900	C	-1.01955100	0.69245300	0.28470900
C	-2.25043400	1.42337900	0.14926500	C	-2.24574700	1.43696700	0.17766000
C	-3.45202600	0.81722700	0.05134500	C	-3.44158600	0.82750100	0.06698400
C	1.30742200	0.71614400	0.12779600	C	1.30849600	0.72368300	0.15063400
C	1.31717400	-0.68141100	0.16141700	C	1.31380600	-0.67358300	0.18834300
C	2.49928200	1.40908700	0.00203000	C	2.50095400	1.41251700	0.00975000
C	2.51248700	-1.37157400	0.07213900	C	2.50568500	-1.36815700	0.08778100
C	3.70913200	0.71853700	-0.08550900	C	3.70748100	0.71760900	-0.08864500
C	3.71647800	-0.67631900	-0.05156400	C	3.71049300	-0.67716100	-0.05096800

O	0.13913900	1.44076500	0.21430800	O	0.14317000	1.45141400	0.24887400
O	0.15170100	-1.41346000	0.29997300	O	0.14748900	-1.40022800	0.34491100
Cl	-4.90835100	1.76897900	-0.09460800	Cl	-4.90324500	1.77329700	-0.07119600
Cl	-4.82031300	-1.41814000	-0.93380100	Cl	-4.76722100	-1.38329000	-1.01234600
Cl	5.18320700	1.63970300	-0.23820600	Cl	5.18296300	1.63338500	-0.25960700
Cl	5.19888200	-1.59055600	-0.15862200	Cl	5.18873200	-1.59667500	-0.17134000
H	-2.17733000	2.50359300	0.14362700	H	-2.17700200	2.51742900	0.18430100
H	-2.19567000	-2.40652300	-0.13484400	H	-2.14981400	-2.40813800	-0.07559900
H	2.50342600	-2.45300100	0.10113200	H	2.49360900	-2.44943200	0.12063900
H	2.48182300	2.49041600	-0.02544900	H	2.48670700	2.49381200	-0.02035800
C	-3.28038500	-1.30281100	1.40774500	C	-3.33166900	-1.31993100	1.39794000
H	-3.31001000	-0.98193300	2.43845300	H	-3.88189600	-2.07318500	1.94044600
**IM7**				**IM8**			
C	-0.31584300	-3.49372600	0.79541500	C	-3.38318000	-0.35335100	-0.00009900
C	-0.21481000	-2.18019900	1.44487700	C	-2.23551100	-1.12950000	0.00106200
C	-0.22588400	-1.04018000	0.72824700	C	-1.00180500	-0.49913000	0.00216100
C	-0.22588400	-1.04018000	-0.72824700	C	-0.92532700	0.89599200	0.00202500
C	-0.21481000	-2.18019900	-1.44487700	C	-2.08206600	1.66088900	0.00075500
C	-0.31584300	-3.49372600	-0.79541500	C	-3.32800400	1.03797200	-0.00022100
C	-0.09983800	1.31684100	-0.69629800	C	1.41131100	0.76787800	0.00189900
C	-0.09983800	1.31684100	0.69629800	C	1.33457100	-0.62502200	0.00214100
C	-0.02177300	2.51394600	-1.39275300	C	2.64479600	1.39614100	0.00056100
C	-0.02177300	2.51394600	1.39275300	C	2.49348600	-1.38292500	0.00101700
C	0.05502600	3.71897700	-0.69975900	C	3.81553800	0.63833800	-0.00044700
C	0.05502600	3.71897700	0.69975900	C	3.73948100	-0.75644300	-0.00021000
O	-0.18057900	0.15169800	-1.41498700	O	0.28013300	1.55809400	0.00322400
O	-0.18057900	0.15169800	1.41498700	O	0.12637400	-1.28689400	0.00365900
Cl	0.56519700	-4.79153800	-1.69073600	Cl	-4.77100000	2.02021600	-0.00169100
Cl	0.56519700	-4.79153800	1.69073600	Cl	-4.96399000	-1.16758800	-0.00128300
Cl	0.15026700	5.20213200	-1.61425300	Cl	5.34743600	1.47331700	-0.00200800
Cl	0.15026700	5.20213200	1.61425300	Cl	5.17168400	-1.75186900	-0.00149500
H	-0.11471600	-2.13556400	-2.52183900	H	-2.00518300	2.73997500	0.00060100
H	-0.11471600	-2.13556400	2.52183900	H	-2.32445700	-2.21133500	0.00103900
H	-0.02175100	2.49908500	2.47440200	H	2.41971700	-2.46227600	0.00118500
H	-0.02175100	2.49908500	-2.47440200	H	2.68937700	2.47697400	0.00038300
C	-1.45890300	-3.89412500	0.00000000	C	-4.45402900	-3.15652900	-0.00155800
H	-2.53268300	-3.77755500	0.00000000	H	-5.53729300	-3.43809100	-0.00266300
**P1**				**P2**			
C	-0.01799500	-3.45548200	0.70257200	C	-3.44448700	-0.68967500	-0.10725900
C	0.20584100	-2.31730600	1.54323300	C	-2.25407200	-1.49140600	-0.06129000
C	-0.19686500	-1.08188400	1.11038500	C	-1.16558400	-1.11164600	0.65755700
C	-0.19686500	-1.08188400	-1.11038500	C	-1.16562100	1.11153700	0.65770500
C	0.20584100	-2.31730600	-1.54323300	C	-2.25405900	1.49138900	-0.06114600
C	-0.01799500	-3.45548200	-0.70257200	C	-3.44449600	0.68967800	-0.10720200

C	0.23154100	1.19086900	-0.70439300	C	1.14485100	0.71194500	0.25808700
C	0.23154100	1.19086900	0.70439300	C	1.14487900	-0.71188800	0.25807500
C	0.12201600	2.39702700	-1.38449500	C	2.36243100	1.37046400	0.07363100
C	0.12201600	2.39702700	1.38449500	C	2.36244900	-1.37044300	0.07374500
C	0.02125000	3.60300100	-0.69898300	C	3.55847000	0.69872300	-0.11552200
C	0.02125000	3.60300100	0.69898300	C	3.55849000	-0.69871000	-0.11544000
O	0.44036500	0.06719000	-1.49524100	O	0.09230600	1.62503300	0.40137400
O	0.44036500	0.06719000	1.49524100	O	0.09229100	-1.62503600	0.40110600
Cl	-0.03265700	-4.99267500	-1.55615500	Cl	-4.92931400	1.57738400	-0.40854100
Cl	-0.03265700	-4.99267500	1.55615500	Cl	-4.92932500	-1.57734700	-0.40868900
Cl	-0.10576900	5.08031600	-1.61561700	Cl	5.02092200	1.62140600	-0.34132700
Cl	-0.10576900	5.08031600	1.61561700	Cl	5.02096700	-1.62138700	-0.34108300
H	0.81403600	-2.44382200	-2.43138500	H	-2.20572100	2.37632500	-0.68551200
H	0.81403600	-2.44382200	2.43138500	H	-2.20576300	-2.37627400	-0.68575500
H	0.12824400	2.38248000	2.46616000	H	2.34526300	-2.45218000	0.07897500
H	0.12824400	2.38248000	-2.46616000	H	2.34525800	2.45220300	0.07878200
C	-1.11678600	-1.03303000	0.00000000	C	-1.14167700	-0.00011700	1.57054800
H	-1.91265700	-1.76870400	0.00000000	H	-0.44049600	-0.00010200	2.39861300
**P3**				**P4**			
C	4.02971600	-0.57401200	0.00007200	C	-3.52554400	1.24923400	-0.00012800
C	1.91630100	-1.98201400	0.00035300	C	-2.19171200	1.57484400	0.00038600
C	0.85912700	-1.03742200	0.00003300	C	-1.06784800	0.68337900	0.00045900
C	0.99283700	0.35729200	0.00010900	C	-1.06776200	-0.68333900	0.00034400
C	2.15455400	1.11568700	0.00021200	C	-2.19192600	-1.57494000	0.00039600
C	3.52481300	0.69683700	0.00000200	C	-3.52523200	-1.24958000	-0.00008200
C	-1.37141200	0.54755000	0.00006000	C	1.29526700	-0.69583600	0.00022800
C	-1.49698500	-0.83848100	-0.00010600	C	1.29525500	0.69562600	0.00033000
C	-2.49571300	1.35457900	0.00014700	C	2.48968500	-1.39349100	0.00000300
C	-2.75471000	-1.42040500	-0.00019200	C	2.48960100	1.39337500	0.00020700
C	-3.76516600	0.77600500	0.00006100	C	3.70055100	-0.69803200	-0.00010200
C	-3.89359200	-0.61567700	-0.00011200	C	3.70052000	0.69800100	-0.00000500
O	-0.13332900	1.15028700	0.00011200	O	0.11669800	-1.40316500	0.00033500
O	-0.39363500	-1.64396400	-0.00018500	O	0.11662200	1.40300200	0.00060600
Cl	4.63256300	2.05716300	-0.00030900	Cl	-4.62825100	-2.62736000	-0.00009200
Cl	5.76212600	-0.83952900	-0.00005700	Cl	-4.62781300	2.62761500	-0.00036700
Cl	-5.15906800	1.82566500	0.00017500	Cl	5.18478300	-1.61463000	-0.00038400
Cl	-5.45769900	-1.38998400	-0.00022500	Cl	5.18469600	1.61468600	-0.00015900
H	1.98708100	2.18339400	0.00034700	H	-1.92158600	-2.62199600	0.00072900
H	1.56683400	-3.00973200	0.00059300	H	-1.92158000	2.62192300	0.00058300
H	-2.84261500	-2.49852500	-0.00032100	H	2.47615200	2.47502000	0.00028800
H	-2.38057200	2.43025200	0.00027700	H	2.47632300	-2.47513800	-0.00008300
C	3.26574600	-1.80609500	0.00036700	C	-4.17570100	0.00013600	-0.00053600
H	3.86721700	-2.70537300	0.00072600	H	-5.25485900	-0.00003400	-0.00102600
**P5**				**P6**			

C	-3.31177600	-0.63980300	-0.00026500	C	-3.45131100	-0.23121600	0.00021100
C	-2.05393100	-1.32612000	-0.00081500	C	-2.25597200	-1.00627700	0.00001100
C	-0.85204900	-0.66748400	-0.00121400	C	-1.01558100	-0.41553000	0.00002200
C	-0.82113500	0.73479200	-0.00096500	C	-0.89306000	0.98171400	-0.00014700
C	-2.01242700	1.44453500	-0.00054600	C	-2.03444700	1.77701300	0.00000000
C	-3.23490600	0.78640400	-0.00020600	C	-3.28510600	1.18833400	0.00008200
C	1.51601500	0.69058500	-0.00080400	C	1.43736400	0.78798700	-0.00018400
C	1.49009600	-0.70537200	-0.00109300	C	1.32104100	-0.60296700	-0.00002100
C	2.72670100	1.36383700	-0.00002300	C	2.68839200	1.38244600	-0.00017000
C	2.67822200	-1.41835600	-0.00062400	C	2.46073900	-1.39109500	0.00009100
C	3.92426000	0.65071700	0.00040400	C	3.83759300	0.59363500	-0.00004700
C	3.89957200	-0.74634900	0.00008900	C	3.72288500	-0.79890400	0.00007600
O	0.35900800	1.43774200	-0.00143600	O	0.32978300	1.60709600	-0.00038300
O	0.31016500	-1.41240200	-0.00190800	O	0.09796300	-1.23127400	-0.00001600
Cl	-4.65371000	1.81394300	-0.00002900	Cl	-4.68376100	2.24744600	0.00024100
Cl	-6.10920100	-1.06713200	0.00170400	Cl	-4.98294200	-2.52126800	-0.00022400
Cl	5.42491100	1.54143600	0.00135700	Cl	5.39238800	1.38630700	-0.00005800
Cl	5.36834900	-1.68850500	0.00058500	Cl	5.12798200	-1.83326800	0.00020400
H	-1.97656400	2.52589500	-0.00050300	H	-1.92624200	2.85352700	0.00011200
H	-2.04204500	-2.40915600	-0.00121900	H	-2.31648800	-2.08613400	0.00021300
H	2.64591500	-2.49967500	-0.00085300	H	2.35866000	-2.46812600	0.00018400
H	2.73062900	2.44562700	0.00021100	H	2.76184200	2.46172200	-0.00026400
C	-4.43846900	-1.48511000	0.00061000	C	-4.72827500	-0.81173000	0.00022100
H	-4.28829000	-2.55469800	0.00035400	H	-5.63766400	-0.23469300	-0.00070000
**P7**				**P8**			
C	-3.51374900	-0.49124400	-0.00008700	C	-3.77520500	-0.49415800	0.25924100
C	-2.31087600	-1.27868000	0.00010400	C	-2.80942600	-1.46875700	0.12179100
C	-1.07931700	-0.55796100	0.00060000	C	-1.48561600	-1.17188400	-0.31550400
C	-1.04901000	0.82679600	0.00058700	C	-1.20935200	0.19340700	-0.60424100
C	-2.22667000	1.56113500	0.00021500	C	-2.17904600	1.16780500	-0.46737500
C	-3.45680500	0.89362800	-0.00013100	C	-3.46616000	0.84561300	-0.03410400
C	1.29245200	0.78879600	0.00060500	C	1.19545700	0.20108300	-0.63592900
C	1.26416400	-0.60572000	0.00057100	C	1.55132600	-1.14018100	-0.46993900
C	2.50559300	1.45669200	0.00024300	C	2.13601400	1.19318800	-0.37833500
C	2.44637900	-1.32610300	0.00022300	C	2.84069600	-1.46427900	-0.05787600
C	3.70054000	0.73733200	-0.00009000	C	3.42527900	0.87127300	0.03000900
C	3.67128500	-0.65907500	-0.00008900	C	3.78113700	-0.47231800	0.19079300
O	0.13694400	1.53447700	0.00105300	O	-0.01215800	0.61465100	-1.14778500
O	0.07631700	-1.30428800	0.00098900	O	0.73183200	-2.20705800	-0.75299700
Cl	-4.90424600	1.87187400	-0.00053600	Cl	-4.64252500	2.11866400	0.12865200
Cl	-5.05250400	-1.30905900	-0.00037400	Cl	-5.37115900	-0.95711500	0.80190500
Cl	5.20436300	1.62199100	-0.00049900	Cl	4.55936100	2.16242100	0.33024300
Cl	5.13575800	-1.60750400	-0.00049000	Cl	5.38168200	-0.94165900	0.70239800
H	-2.18278700	2.64129400	0.00016400	H	-1.92988100	2.19079400	-0.71579300

H	-1.36974700	-3.21383100	0.00013800	H	-3.06624500	-2.49550700	0.34969000
H	2.40976400	-2.40727500	0.00021500	H	3.10145700	-2.50773200	0.05795800
H	2.51503000	2.53840800	0.00025500	H	1.84975800	2.22669900	-0.51988500
C	-2.30532600	-2.67543900	-0.00020100	C	-0.59470300	-2.24535900	-0.46350000
H	-3.22762000	-3.23517900	-0.00011600	H	-0.94000100	-3.26688100	-0.39026500
**TS1**				**TS2**			
C	-3.42392100	-0.73293900	-0.09409400	C	3.47108100	0.72152600	-0.03612000
C	-2.34224100	-1.43965700	0.28037600	C	2.33844400	1.44320500	0.17431100
C	-1.14910500	-0.78215200	0.83653800	C	1.14215800	0.87241300	0.73102700
C	-1.14909200	0.78214800	0.83653100	C	1.14215900	-0.87241400	0.73102000
C	-2.34223100	1.43965300	0.28037300	C	2.33845900	-1.44321000	0.17433400
C	-3.42391600	0.73293900	-0.09409400	C	3.47109000	-0.72152100	-0.03610100
C	1.16533600	0.70061700	0.28527200	C	-1.16630600	-0.70294100	0.19322200
C	1.16533500	-0.70062000	0.28527000	C	-1.16631000	0.70294400	0.19324000
C	2.35616400	1.38536100	0.06494700	C	-2.36918600	-1.38330600	0.03223600
C	2.35616400	-1.38536300	0.06494100	C	-2.36919500	1.38330500	0.03227200
C	3.54198700	0.69927700	-0.16685100	C	-3.56515400	-0.69925500	-0.14478600
C	3.54198700	-0.69927700	-0.16685500	C	-3.56515900	0.69925100	-0.14476700
O	0.04299700	1.46928100	0.48290900	O	-0.04257800	-1.49508700	0.31251600
O	0.04299500	-1.46928600	0.48292800	O	-0.04258900	1.49509600	0.31256200
Cl	-4.83419400	1.58732500	-0.67625000	Cl	4.91285400	-1.58351200	-0.53067100
Cl	-4.83420300	-1.58731700	-0.67625600	Cl	4.91282500	1.58352200	-0.53074000
Cl	5.00156300	1.61747500	-0.44690500	Cl	-5.03426600	-1.61747600	-0.35502400
Cl	5.00156300	-1.61747400	-0.44691500	Cl	-5.03427800	1.61746700	-0.35498000
H	-2.30878100	2.51642000	0.16689900	H	2.29166800	-2.48611600	-0.11742900
H	-2.30879400	-2.51642500	0.16690400	H	2.29163400	2.48610000	-0.11748900
H	2.34159200	-2.46717300	0.07055300	H	-2.35324200	2.46510100	0.03679200
H	2.34159100	2.46717200	0.07056300	H	-2.35322400	-2.46510200	0.03672700
C	-1.17357700	0.00000000	2.06207700	C	1.07965800	-0.00000700	1.88388100
H	-1.18127300	-0.00003000	3.13931700	H	0.24277400	-0.00000600	2.57220800
**TS3**				**TS4**			
C	3.48764200	0.73343100	0.01187200	C	-3.49156300	-0.68740600	-0.07181000
C	2.34927700	1.46491200	0.08183300	C	-2.30950800	-1.50570000	-0.26489300
C	1.09042900	0.82887300	0.41929600	C	-1.13259200	-1.13040700	0.27738600
C	1.09042700	-0.82888000	0.41929300	C	-1.13257300	1.13038600	0.27737100
C	2.34927600	-1.46491100	0.08182900	C	-2.30949500	1.50568300	-0.26490200
C	3.48764100	-0.73342900	0.01187300	C	-3.49155600	0.68740800	-0.07181500
C	-1.20005600	-0.69958400	-0.09920700	C	1.17708000	0.70943200	-0.08745600
C	-1.20005400	0.69957800	-0.09920600	C	1.17708500	-0.70944300	-0.08746300
C	-2.40217800	-1.39333600	-0.08905200	C	2.40093800	1.37510200	-0.05475100
C	-2.40217500	1.39333300	-0.08905000	C	2.40094700	-1.37510600	-0.05477300
C	-3.60927800	-0.69984800	-0.08720400	C	3.61230600	0.69852300	-0.03921000
C	-3.60927600	0.69984900	-0.08720300	C	3.61231100	-0.69852100	-0.03922400
O	-0.02943600	-1.41268500	-0.19932600	O	0.08971100	1.57595500	-0.18615800

O	-0.02943300	1.41267600	-0.19933100	O	0.08971300	-1.57597700	-0.18610400
Cl	4.99644300	-1.57737500	-0.25294600	Cl	-5.00649700	1.57839400	-0.03418200
Cl	4.99644300	1.57737600	-0.25295100	Cl	-5.00651300	-1.57837600	-0.03416900
Cl	-5.09643300	-1.61338600	-0.07964400	Cl	5.09243500	1.61956100	-0.01092200
Cl	-5.09642900	1.61339000	-0.07964300	Cl	5.09244600	-1.61955000	-0.01095400
H	2.35764500	-2.52563100	-0.13762200	H	-2.37208400	2.34962900	-0.94286300
H	2.35764200	2.52563200	-0.13761600	H	-2.37210200	-2.34964600	-0.94285400
H	-2.38939100	2.47495600	-0.09533900	H	2.38393200	-2.45683100	-0.05366200
H	-2.38939800	-2.47495800	-0.09534100	H	2.38391800	2.45682600	-0.05363300
C	0.94820200	0.00000600	1.61280500	C	-1.07702400	-0.00000200	1.18792400
H	1.65478400	0.00000400	2.43603500	H	-0.99901300	0.00000700	2.26665400
**TS5**				**TS6**			
C	-3.60656900	-0.59679200	0.06009200	C	-3.66914200	-0.60373400	0.03020300
C	-2.38810800	-1.41044000	0.32167100	C	-2.60967300	-1.52171500	0.35922100
C	-1.02505900	-0.67321000	0.29026500	C	-0.99161800	-0.75387400	0.45562000
C	-1.09291800	0.78716600	0.07472200	C	-1.08756900	0.67213400	0.23158100
C	-2.25864400	1.44822700	-0.04708600	C	-2.25211400	1.37477300	0.23717100
C	-3.52580900	0.74311500	-0.07148800	C	-3.52660000	0.75219400	0.11965100
C	1.25687200	0.73683500	-0.00336500	C	1.26390600	0.69186800	0.00093000
C	1.24210800	-0.64269300	-0.23051000	C	1.29096700	-0.70029900	-0.07221000
C	2.46019500	1.41114300	0.14310300	C	2.44789700	1.41401200	0.01697500
C	2.43967200	-1.33979700	-0.31383400	C	2.50299400	-1.37184000	-0.12671300
C	3.66158800	0.71391400	0.05111800	C	3.66788300	0.74545300	-0.04992000
C	3.65130600	-0.66584600	-0.18360100	C	3.69559200	-0.65173000	-0.12646500
O	0.09316600	1.47047700	0.03876000	O	0.07548400	1.37742300	0.00756200
O	0.05430100	-1.30401400	-0.40342800	O	0.11783100	-1.41675300	-0.10537400
Cl	-4.94118200	1.73523800	-0.33370500	Cl	-4.88847800	1.82690600	-0.11368300
Cl	-5.09020400	-1.49260700	-0.10442100	Cl	-5.12130600	-1.34251800	-0.61506200
Cl	5.15523100	1.59977600	0.23405600	Cl	5.13608000	1.68894000	-0.03577800
Cl	5.13269000	-1.58151800	-0.31028300	Cl	5.19998900	-1.53231000	-0.21535500
H	-2.25854600	2.52439500	-0.15592700	H	-2.19663300	2.45432300	0.18835800
H	-2.40111800	-2.40036700	-0.13751000	H	-2.66872500	-2.49479400	-0.12553100
H	2.41972400	-2.40679800	-0.49107100	H	2.51160900	-2.45213000	-0.18137300
H	2.45495800	2.47880600	0.31706600	H	2.41346000	2.49379500	0.07194200
C	-1.54268700	-1.25611700	1.50659800	C	-1.70549900	-1.42924500	1.50099400
H	-1.38752700	-1.49588100	2.54266800	H	-1.31524100	-2.29185800	2.02479300
**TS7**				**TS8**			
C	-3.59192100	-0.65089700	0.14830600	C	-3.68163600	-0.59037900	0.16999300
C	-2.24913500	-1.44618300	0.36674400	C	-2.20135600	-1.52461200	0.59582100
C	-1.00268300	-0.66061000	0.33774500	C	-1.00977400	-0.72735800	0.60556100
C	-1.01983000	0.68008900	0.25228600	C	-1.01943800	0.63247800	0.61382800
C	-2.24681200	1.42722900	0.15352200	C	-2.19238300	1.38848100	0.36476700
C	-3.44869600	0.82364800	0.06376300	C	-3.38883600	0.82780300	0.03756200
C	1.30973300	0.71805700	0.12969800	C	1.30749100	0.67439500	0.40493100

C	1.31925300	-0.67931700	0.16775400	C	1.30854600	-0.71669100	0.28400300
C	2.50173700	1.41015500	0.00039500	C	2.48471800	1.38310100	0.23374900
C	2.51407300	-1.37033500	0.07884600	C	2.48208400	-1.39023200	-0.00598200
C	3.71122900	0.71883600	-0.08634400	C	3.67355400	0.70936300	-0.04935500
C	3.71816100	-0.67587600	-0.04840200	C	3.67238300	-0.68112100	-0.17204500
O	0.14192800	1.44272800	0.21637900	O	0.15696100	1.37090300	0.69555900
O	0.15346500	-1.40986900	0.31112100	O	0.16054100	-1.45656000	0.48376200
Cl	-4.90669800	1.77677400	-0.05855500	Cl	-4.66903800	1.87447500	-0.54591500
Cl	-4.81381000	-1.39238200	-0.97544800	Cl	-4.85811700	-1.31568300	-0.96198500
Cl	5.18556700	1.63905500	-0.24313000	Cl	5.13370000	1.64523800	-0.24513400
Cl	5.20022300	-1.59104500	-0.15430200	Cl	5.12973600	-1.57304300	-0.52871800
H	-2.17259400	2.50739200	0.15208600	H	-2.08488600	2.46533700	0.33620200
H	-2.17013800	-2.40729500	-0.14448400	H	-2.07553600	-2.51473700	0.15724500
H	2.50468900	-2.45165800	0.11126100	H	2.46817100	-2.46832200	-0.09429000
H	2.48468900	2.49140600	-0.03002800	H	2.47292300	2.46087200	0.32633500
C	-3.29344400	-1.32125000	1.38041000	C	-3.40457000	-1.30521900	1.38212100
H	-3.64957600	-1.59482400	2.35720700	H	-4.02217000	-2.06472000	1.83998300
**TS9**				**TS10**			
C	-3.51062300	0.57633100	0.08874100	C	3.49951300	0.58804700	-0.14435600
C	-2.28280000	1.40323700	0.01521700	C	2.28432200	1.30449800	-0.19655900
C	-1.04661100	0.60534800	0.13902600	C	1.07161300	0.60888200	-0.23139600
C	-1.04508500	-0.76223200	0.08487500	C	1.06510800	-0.78782600	-0.15036200
C	-2.24602200	-1.49281900	0.01880300	C	2.25676000	-1.47861000	-0.07013900
C	-3.46152300	-0.82111200	0.01668600	C	3.48066300	-0.79988100	-0.06824900
C	1.29336200	-0.75285000	0.08772300	C	-1.27256200	-0.76136900	-0.11363000
C	1.28150100	0.64169200	0.14146200	C	-1.25824400	0.62758900	-0.22635100
C	2.49977600	-1.42815400	0.01281200	C	-2.47671800	-1.43167700	0.01806300
C	2.46614100	1.35473000	0.11834400	C	-2.44040000	1.34479000	-0.20715600
C	3.70005700	-0.71704200	-0.00575600	C	-3.67445300	-0.71742900	0.03296200
C	3.68445600	0.67872800	0.04649600	C	-3.65727800	0.67524600	-0.08031400
O	0.13144500	-1.49064900	0.11842300	O	-0.11110900	-1.50086400	-0.14177000
O	0.09420700	1.34745800	0.22937800	O	-0.07307000	1.32569400	-0.37775900
Cl	-4.92976300	-1.76559400	-0.06411600	Cl	4.94721600	-1.73392600	0.03090100
Cl	-4.99835900	1.43539500	0.30985900	Cl	4.98239200	1.48859200	-0.18798100
Cl	5.19421300	-1.61211400	-0.09597200	Cl	-5.16771700	-1.60317300	0.19627600
Cl	5.15605900	1.61426500	0.02427800	Cl	-5.12662300	1.61411800	-0.06588500
H	-2.20539600	-2.57068400	-0.05174600	H	2.23508000	-2.55798200	0.00434900
H	-2.28170000	2.28945500	0.64978900	H	2.28507300	2.36581000	-0.40089100
H	2.43688900	2.43542600	0.15829800	H	-2.41030900	2.42277400	-0.29168600
H	2.50085800	-2.50914800	-0.02781200	H	-2.47754100	-2.50966100	0.10765200
C	-2.48953700	1.73414200	-1.46962700	C	2.02761600	1.38161400	1.98356000
H	-2.49097800	0.95729800	-2.23858800	H	2.60593500	2.34178100	1.91407700
**TS11**				**TS12**			
C	-0.33200900	-3.49218400	0.80132200	C	-3.39734900	-0.56016700	-0.02520200

C	-0.21134200	-2.17651100	1.44528400	C	-2.19620900	-1.28648700	-0.11685000
C	-0.20303800	-1.03762900	0.72742600	C	-0.98823700	-0.61911100	-0.07469600
C	-0.20303800	-1.03762900	-0.72742600	C	-0.95750200	0.77783200	-0.02563900
C	-0.21134200	-2.17651100	-1.44528400	C	-2.14383400	1.50215000	-0.03428900
C	-0.33200900	-3.49218400	-0.80132200	C	-3.36707800	0.85063600	-0.07355100
C	-0.07298600	1.31953100	-0.69626000	C	1.38093800	0.72739200	-0.00160200
C	-0.07298600	1.31953100	0.69626000	C	1.35185300	-0.66702600	-0.05811100
C	-0.01122200	2.51751800	-1.39256800	C	2.59384700	1.39470800	0.04405000
C	-0.01122200	2.51751800	1.39256800	C	2.53620100	-1.38496300	-0.06935000
C	0.05038500	3.72364300	-0.69960600	C	3.78903900	0.67704400	0.03229200
C	0.05038500	3.72364300	0.69960600	C	3.76011400	-0.71850800	-0.02461100
O	-0.13392200	0.15319900	-1.41530900	O	0.22675200	1.47852400	0.00934800
O	-0.13392200	0.15319900	1.41530900	O	0.16772500	-1.36947300	-0.10421100
Cl	0.54966100	-4.79274800	-1.70463500	Cl	-4.82917400	1.78608600	-0.09031800
Cl	0.54966100	-4.79274800	1.70463500	Cl	-4.90108000	-1.44397800	-0.45550500
Cl	0.12595600	5.20791400	-1.61444800	Cl	5.29223700	1.56113700	0.08998400
Cl	0.12595600	5.20791400	1.61444800	Cl	5.22516800	-1.66587300	-0.04128400
H	-0.10609400	-2.13204300	-2.52189500	H	-2.10108400	2.58313800	-0.01395800
H	-0.10609400	-2.13204300	2.52189500	H	-2.20685900	-2.36617300	-0.17814100
H	-0.01124900	2.50270500	2.47421800	H	2.49993300	-2.46529100	-0.11298200
H	-0.01124900	2.50270500	-2.47421800	H	2.60186500	2.47556900	0.08800100
C	-1.45642600	-3.89512900	0.00000000	C	-4.56621700	-1.18257300	1.70133600
H	-2.49245700	-4.18978100	0.00000000	H	-4.10464400	-2.20053100	1.78441500
**TS13**				**TS14**			
C	3.34194900	-0.44356600	0.36763800	C	-3.51252600	-0.67983000	0.37955200
C	2.14370700	-1.17341900	0.39325600	C	-2.22795100	-1.37623700	0.44294600
C	0.91683000	-0.54635800	0.25854800	C	-1.04097200	-0.68351300	0.33645300
C	0.85805700	0.83757300	0.08789500	C	-1.05091100	0.70234200	0.13372400
C	2.02491000	1.58600900	0.05759900	C	-2.25743600	1.43085200	0.08625400
C	3.25237500	0.94543400	0.19569000	C	-3.44677200	0.78211300	0.23696700
C	-1.47715100	0.72619800	-0.01918900	C	1.28445400	0.70256400	0.02064700
C	-1.41943200	-0.65805000	0.15093200	C	1.29704500	-0.67842900	0.20281500
C	-2.70078500	1.35834700	-0.16130600	C	2.46486600	1.40350900	-0.14501500
C	-2.58809900	-1.40110000	0.17689800	C	2.50191100	-1.36011900	0.21947000
C	-3.88036900	0.61500500	-0.13549600	C	3.68199700	0.72313900	-0.12815200
C	-3.82351300	-0.77012500	0.03445800	C	3.69894400	-0.66350200	0.05397600
O	-0.33863400	1.50304600	-0.05052200	O	0.09467600	1.41319500	-0.00412500
O	-0.22341800	-1.32337500	0.29624700	O	0.13531700	-1.39515600	0.37678300
Cl	4.70624500	1.92385800	0.16972700	Cl	-4.92669100	1.65788200	0.30231200
Cl	5.54225700	-1.63561100	-0.84148500	Cl	-4.36709500	-1.29185900	-1.54362000
Cl	-5.39871000	1.45527500	-0.31866000	Cl	5.14991700	1.63723200	-0.33897200
Cl	-5.26755400	-1.74877000	0.07356100	Cl	5.19122200	-1.56102400	0.08048400
H	1.96372200	2.65841200	-0.07102200	H	-2.20854900	2.50485300	-0.03583400
H	2.16684500	-2.24878000	0.51941400	H	-2.21652300	-2.45587000	0.48354500

H	-2.53048300	-2.47332900	0.30872100	H	2.50368900	-2.43263600	0.36049400
H	-2.73003700	2.43175900	-0.29235100	H	2.43387600	2.47554200	-0.28668900
C	4.62466900	-1.13874300	0.53717100	C	-4.03514700	-1.21686500	1.55525000
H	5.08943300	-1.33943700	1.49144700	H	-4.30244100	-2.27028800	1.62444500
**TS15**				**TS16**			
C	-3.52395400	-0.69231600	0.28816200	C	-3.39419700	-0.57311700	-0.01040900
C	-2.20555400	-1.35591100	0.35782800	C	-2.18730800	-1.29676000	-0.09592600
C	-1.03830100	-0.67399900	0.24061000	C	-0.98558300	-0.62842100	-0.07226400
C	-1.02917200	0.74622600	0.09012200	C	-0.95532500	0.77401300	-0.02546100
C	-2.21016900	1.44884100	0.08179200	C	-2.13990700	1.49452000	-0.02404600
C	-3.43398600	0.77266200	0.18827900	C	-3.36341200	0.83528100	-0.06154900
C	1.31356800	0.71629800	0.01044300	C	1.38392600	0.72444000	-0.00520100
C	1.31022300	-0.66899400	0.16020600	C	1.35589300	-0.66991100	-0.05455800
C	2.50838800	1.40292400	-0.12823300	C	2.59555400	1.39421600	0.03668100
C	2.50743100	-1.36680900	0.17189500	C	2.54137200	-1.38643400	-0.06155000
C	3.71541700	0.70703500	-0.11601800	C	3.79149200	0.67802500	0.02916000
C	3.71429200	-0.68367800	0.03506200	C	3.76418800	-0.71801700	-0.02077900
O	0.14129900	1.44218600	0.00096000	O	0.22804600	1.47379300	0.00158700
O	0.14158000	-1.37901100	0.30349100	O	0.17323700	-1.37324300	-0.09918000
Cl	-4.89891800	1.68888900	0.00799900	Cl	-4.82648400	1.78508500	-0.10028000
Cl	-4.56472900	-1.57432400	-1.14239300	Cl	-4.89821500	-1.44733400	-0.46702900
Cl	5.19821100	1.60748800	-0.29262900	Cl	5.29368900	1.56418500	0.08166000
Cl	5.19659000	-1.60137100	0.05593900	Cl	5.23045400	-1.66328200	-0.03190300
H	-2.18995600	2.52657800	-0.01016600	H	-2.10068000	2.57563000	-0.00765800
H	-2.19343700	-2.42832900	0.50058500	H	-2.20188100	-2.37754900	-0.11167900
H	2.49320200	-2.44230700	0.28739600	H	2.50624500	-2.46702400	-0.09910000
H	2.49313900	2.47833200	-0.24445000	H	2.60211400	2.47529600	0.07487900
C	-4.10850000	-0.63454600	1.58302800	C	-4.48289300	-1.38895600	1.68366900
H	-3.81369500	-0.99765000	2.56040700	H	-5.14942400	-0.50113000	1.81609500
**TS17**				**TS18**			
C	-3.52036600	-0.69749300	0.26325700	C	-3.51564500	-0.69338400	0.36006800
C	-2.21724500	-1.36856500	0.34425900	C	-2.19887200	-1.36043100	0.44338400
C	-1.02196800	-0.67665300	0.21693200	C	-1.03583000	-0.68702100	0.28752500
C	-1.02350200	0.70186700	0.03837500	C	-1.03349400	0.73371500	0.12013600
C	-2.23250300	1.42384700	0.03792900	C	-2.21254900	1.44086800	0.11232000
C	-3.42781100	0.77521500	0.17290200	C	-3.43522200	0.76785100	0.23124400
C	1.31117900	0.70354300	-0.03155400	C	1.30890700	0.70708600	0.01617100
C	1.31620000	-0.67980200	0.14796800	C	1.31200600	-0.67513600	0.18596400
C	2.50087300	1.39614200	-0.16823200	C	2.49706200	1.40012700	-0.13979200
C	2.51849900	-1.36502600	0.19206500	C	2.51323000	-1.36593900	0.19943800
C	3.71503500	0.71099500	-0.12478400	C	3.70816900	0.71113700	-0.12619500
C	3.72291300	-0.67450100	0.05621300	C	3.71507300	-0.67746100	0.04466400
O	0.13077000	1.42339300	-0.07559800	O	0.12948300	1.42694800	0.00482200
O	0.15032400	-1.39931700	0.28688800	O	0.14779800	-1.38706800	0.34332600

Cl	-4.90566500	1.67409300	0.26078800	Cl	-4.89009300	1.68245400	-0.02673100
Cl	-4.60700100	-1.39150100	-1.29510500	Cl	-4.48005100	-1.59802600	-1.22099200
Cl	5.19161700	1.62127800	-0.30081000	Cl	5.18444300	1.61677100	-0.32550400
Cl	5.21121800	-1.58013900	0.11680300	Cl	5.20220100	-1.58612900	0.06820300
H	-2.19131700	2.50245200	-0.04365300	H	-2.18770200	2.51560200	-0.00888500
H	-2.20796300	-2.44634100	0.42263900	H	-2.19315000	-2.42547000	0.63120200
H	2.51218700	-2.43785100	0.33091500	H	2.50603300	-2.43975000	0.33011200
H	2.47874600	2.46869400	-0.30771500	H	2.47444000	2.47370900	-0.27075200
C	-3.91957200	-1.10905200	1.55116500	C	-4.13362400	-0.66388200	1.61492500
H	-4.30366200	-0.62607900	2.44005500	H	-5.03364700	-0.22449500	2.01945200
**TS19**				**TS20**			
C	3.50407700	0.46455800	-0.08560700	C	3.54498000	0.52865900	-0.06571900
C	2.30572700	1.18279800	-0.10861100	C	2.29674700	1.39308500	0.07544500
C	1.07669700	0.53754800	-0.06492800	C	1.04486900	0.57587800	-0.07324700
C	1.04479300	-0.85823800	0.00934100	C	1.03936200	-0.79074100	0.01077500
C	2.22489200	-1.58142700	0.03949400	C	2.23045900	-1.51554000	0.09958700
C	3.45723300	-0.92830300	-0.01022000	C	3.47293800	-0.83127400	0.01918000
C	-1.29485700	-0.80016800	0.01818000	C	-1.29982700	-0.76826100	-0.02192200
C	-1.25818600	0.59118100	-0.06009900	C	-1.28096700	0.62356300	-0.12909800
C	-2.51035900	-1.46118600	0.06114900	C	-2.51139100	-1.43470500	0.05487700
C	-2.43535500	1.31843300	-0.09504900	C	-2.46274800	1.34169600	-0.15762000
C	-3.70083700	-0.73561900	0.02553900	C	-3.70817400	-0.71866200	0.02163500
C	-3.66347900	0.65903800	-0.05277200	C	-3.68515700	0.67399500	-0.08519100
O	-0.14054800	-1.55489800	0.05555600	O	-0.14291100	-1.51229300	-0.00490400
O	-0.06692900	1.28651800	-0.10381500	O	-0.08931100	1.31939400	-0.21345600
Cl	4.90962600	-1.89284800	0.02217600	Cl	4.91225600	-1.82268500	-0.04033700
Cl	5.01412100	1.32406400	-0.16037500	Cl	5.03379600	1.38591900	-0.31594900
Cl	-5.20914600	-1.60972200	0.08086700	Cl	-5.20840600	-1.60371900	0.11633700
Cl	-5.12237900	1.61277800	-0.09894600	Cl	-5.15299000	1.61558900	-0.12959300
H	2.17967800	-2.66089200	0.09677900	H	2.20189700	-2.59120100	0.19349200
H	2.29056300	2.32383200	-0.43201000	H	2.31660700	2.18312700	-0.69786600
H	-2.38903200	2.39770700	-0.15368700	H	-2.42772900	2.42003800	-0.23654900
H	-2.52579000	-2.54112200	0.12185500	H	-2.51855000	-2.51324900	0.13762500
C	2.27685100	3.19246800	0.64573800	C	2.33246600	2.03335600	1.42959000
H	2.87343700	2.75839100	1.47693900	H	3.26505800	2.60142000	1.56265500
**TS21**							
C	-3.53546400	-0.61584300	-0.09348700				
C	-2.44768000	-1.35446800	0.27982000				
C	-1.21771300	-0.72567800	0.78469200				
C	-1.14571600	0.72317400	0.51013900				
C	-2.23972000	1.44624400	0.12604700				
C	-3.46085800	0.80199800	-0.14609200				
C	1.18757500	0.66293200	0.38460200				
C	1.17877500	-0.70867600	0.12717100				

C	2.37486400	1.37595300	0.31725100	
C	2.35787900	-1.35625500	-0.21223400	
C	3.56017600	0.72910200	-0.01971200	
C	3.55026600	-0.64268800	-0.29475100	
O	0.04091900	1.36275400	0.68751300	
O	0.02932300	-1.47018500	0.22459300	
Cl	-4.82347400	1.76861500	-0.59976700	
Cl	-5.02004600	-1.44041600	-0.50854200	
Cl	5.02970800	1.66667000	-0.09654300	
Cl	5.00646400	-1.49908200	-0.73232400	
H	-2.15317600	2.51808400	0.00496500	
H	-2.50178100	-2.43444400	0.31912800	
H	2.34154700	-2.42144500	-0.39974500	
H	2.36637000	2.43778800	0.52355100	
C	-0.63444200	-1.23892600	2.06701100	
H	-1.05762000	-2.25013200	2.20447300	
